# Role of the circadian clock in the statistics of locomotor activity in Drosophila

**DOI:** 10.1371/journal.pone.0202505

**Published:** 2018-08-23

**Authors:** Guadalupe Cascallares, Sabrina Riva, D. Lorena Franco, Sebastian Risau-Gusman, Pablo M. Gleiser

**Affiliations:** 1 Statistical and Interdisciplinary Physics Group, Centro Atómico Bariloche, Bariloche, Río Negro, Argentina; 2 Medical Physics Department, CONICET and Centro Atómico Bariloche, Av. E. Bustillo 9500, (8400) San Carlos de Bariloche, Río Negro, Argentina; University of Lübeck, GERMANY

## Abstract

In many animals the circadian rhythm of locomotor activity is controlled by an endogenous circadian clock. Using custom made housing and video tracking software in order to obtain high spatial and temporal resolution, we studied the statistical properties of the locomotor activity of wild type and two clock mutants of *Drosophila melanogaster*. We show here that the distributions of activity and quiescence bouts for the clock mutants in light-dark conditions (*LD*) are very different from the distributions obtained when there are no external cues from the environment (*DD*). In the wild type these distributions are very similar, showing that the clock controls this aspect of behavior in both regimes (*LD* and *DD*). Furthermore, the distributions are very similar to those reported for Wistar rats. For the timing of events we also observe important differences, quantified by how the event rate distributions scale for increasing time windows. We find that for the wild type these distributions can be rescaled by the same function in *DD* as in *LD*. Interestingly, the same function has been shown to rescale the rate distributions in Wistar rats. On the other hand, for the clock mutants it is not possible to rescale the rate distributions, which might indicate that the extent of circadian control depends on the statistical properties of activity and quiescence.

## Introduction

Circadian rhythms are daily oscillations of physiological and behavioral processes, which are present in all animals, including humans. These rhythms can be entrained by cycles of light or temperature, and the fact that they persist under constant environmental conditions shows that they are endogenous. In fact, research has shown that they are generated by the coherent interaction of cellular clocks [1–5].

The presence of a circadian clock has been shown to provide an increase of fitness in cyanobacteria [6, 7], fruit flies [8], plants [9], and even mammals [10]. This is probably the case for most species, since there is a close relationship between circadian rhythms and many metabolic processes, and therefore many behaviors. As a consequence, disruption of the biological clock can accelerate, or even cause many diseases [11, 12], and conversely, taking the circadian rhythms into account can lead to more effective treatments for some diseases [13, 14]. Thus, it is important not only to advance further in the characterization of the relation between circadian clocks and behavioral as well as metabolic systems in which the circadian clock and behavioral as well as metabolic systems interact, but also to characterize these interactions with as much detail as possible.

Since the discovery of the first circadian clock mutants in the fruit fly by Konopka and Benzer [15], model organisms have proven to be essential to understand how cellular clocks work. Specially because the main functional components of such clocks are conserved across most species. Furthermore, the discovery that complex behavioral traits that are common in mammals, such as sleep and movement patterns, can also be found in relatively simple organisms [16, 17], suggests that the study of these organisms can also help us to understand the relationship between the circadian clock and complex behavior [18].

For most animal models the main behavioral output used to study the circadian clock is locomotor activity. However, until very recently, activity was monitored only in very controlled artificial settings, such as a running-wheel for mice and hamsters and very small tubes in *Drosophila melanogaster* [19, 20]. In fruit flies the most widely used system is the Drosophila Activity Monitoring (DAM) System [20], in which activity is registered as the number of times that the fly inside a glass tube crosses an infrared beam in a given time period. In the last years, there has been an increasing interest in studying the influence of the circadian clock on behavioral traits under natural, or at least less artificial conditions [21, 22]. This concern has been extended to analyzing activity in less confined settings, which has been made possible by new tools that use video tracking software [23–25] on custom made arenas [26].

Increasing the spatial and temporal resolution in the analysis of the spontaneous behavior of isolated insects allows for the quantification of new statistical properties. In *Drosophila* the distribution of time intervals of quiescence is broad and in some cases can be fit by a power law function, presenting a self-similar temporal pattern [27]. In contrast the distribution of time intervals of activity presents a clear exponential, indicating a characteristic time scale in the intervals between consecutive occurrences [28].

An intriguing aspect is the role that the circadian clock plays in the activity and quiescence distributions, given that these distributions seem to involve many time scales in one case and a characteristic time scale in another. Furthermore, because locomotor activity is a complex behavior, even in fruit flies, it is important to understand what is the role of the clock in the control of different aspects of locomotor activity, that can be quantified by suitable statistical analysis. In order to tackle these questions here we have analyzed several features of the locomotor activity of wild type and clock mutant flies, using a custom build arena and video tracking software to record their activity under both cyclic and constant environmental conditions.

## Materials and methods

We have performed two series of experiments using four different strains of *Drosophila melanogaster*: Canton-S (hereafter referred to as “wild type”), and two clock mutants: *per*^01^ (*yw* background), *pdf*^01^ (*yw* background). Also, as a background control we analyzed the behavior of *yw* flies. Each experiment involved 10 − 25 male flies selected 2 − 3 days after eclosion. For each experiment the flies were enclosed in an array of transparent tracks (each track measures 10(*width*) × 7(*height*) × 100(*length*) mm^3^), one fly per track. One end of the track was sealed and contained approximately 1 *cm*^3^ of fly food medium containing banana jaggery media, as in [29], whereas the other end was closed with a cotton plug. The flies were exposed to two different light conditions: a 24 hour cycle (called LD) alternating light (12 hours, with 200 lux of white leds light) and darkness (12 hours, infrared led light, 850 nm.) and a regime of “constant darkness” (DD) with only infrared illumination. Each experiment consisted of two phases: 6 days in LD conditions (entrainment phase) followed by 6 days in DD conditions. All experiments were conducted at 22 − 24°C, and ≈ 50% humidity.

To register the activity of the flies we have used a custom built box for the fly tracks, where the temperature and humidity can be controlled. Webcams were attached to the box allow us to register fly activity, both during subjective day and subjective night. The distance between the cameras and the tracks was 30*cm* allowing us to obtain a resolution between 2.5 and 3 pixels per *mm*. The video signal is fed to a computer where we use the software Pysolo [24] to record the activity of each individual fly in real time. The tracking software gives one reading per second of the position of the fly, and these readings are saved to a text file. Subtracting results from consecutive readings this can be transformed to a discrete time series where each value represents the displacement of the fly during one second. All statistical analysis was performed using custom build programs including C-shell scripts and Fortran 95 programs. In order to obtain meaningful statistics a minimum level of activity was required. As not all the flies reached this level, specially in *DD*, we were forced to discard the data in some of the flies.

## Results

**Fig 1:**
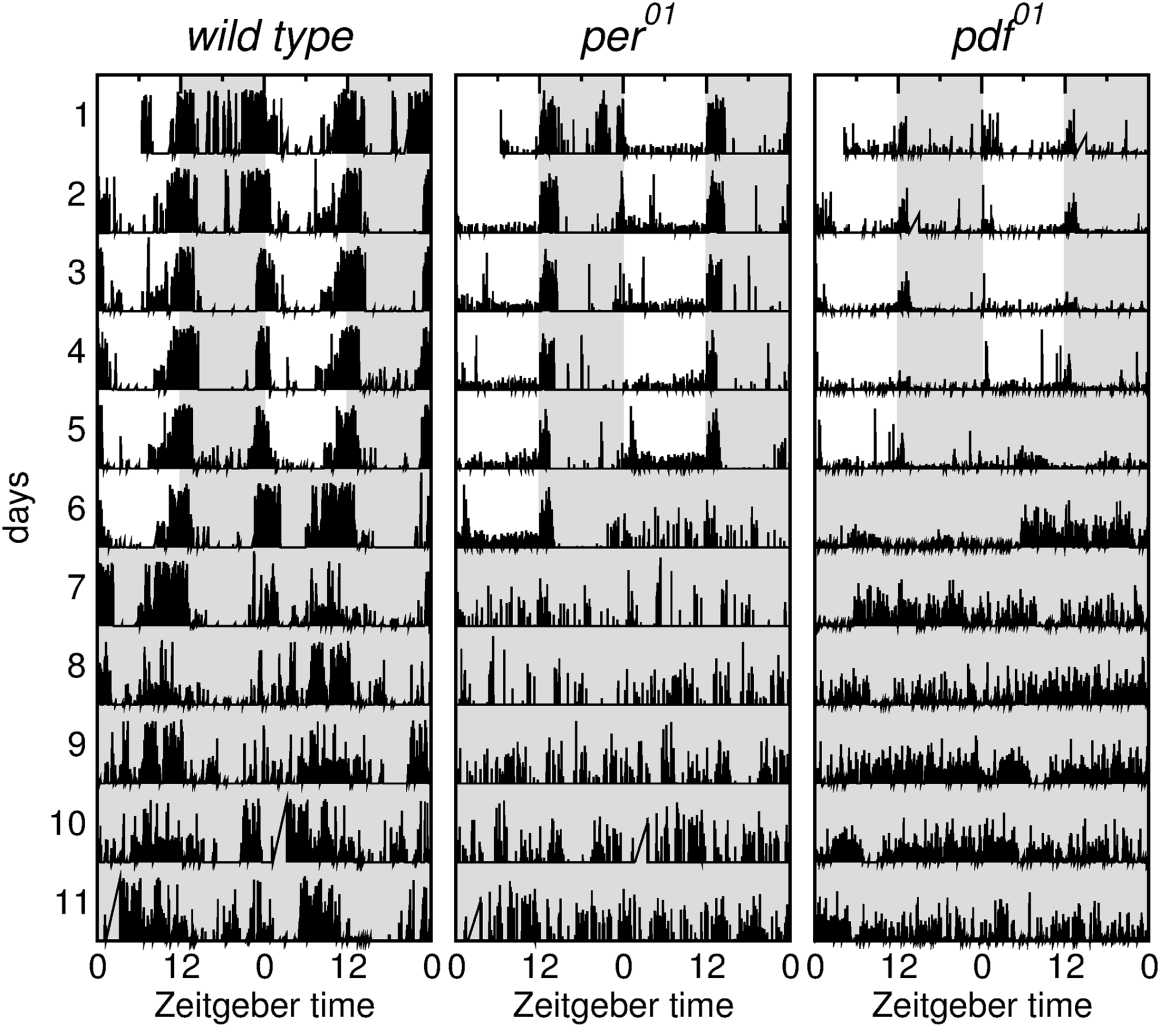
Activity plots for wild type, *per*^01^ and *pdf*^01^ mutants. Distance as a function of time presented as double plotted activity plots for wild type (left), *per*^01^ (center), *pdf*^01^ (right). The plots show both *LD* and *DD* conditions.

In order to visualize the activity of wild type, *per*^01^ and *pdf*^01^ mutant flies we show in Fig 1 three double plotted activity plots for individual flies, where consecutive days are aligned horizontally in each plot (S2 Fig shows the average activity plots for all wild type, *per*^01^ and *pdf*^01^ mutants). The height of the vertical bars indicates the distance traveled per second. Flies are entrained during six days in *LD* conditions, given by 12 hours of light (white background) and 12 hours of darkness (gray background). Then, the lights are turned off, and the flies freerun in constant *DD* conditions for another six days. The leftmost column in Fig 1 shows the activity of a wild type fly. Two daily peaks of activity can be clearly seen as the fly becomes entrained with the light-dark cycle. Note how the activity peaks anticipate the morning and the evening, and also that the fly is mostly inactive during the night. When the lights are turned off the flies maintain their rhythmicity. Note that even when the two peaks are now not clearly divided still the activity is concentrated in the subjective day and there is quantitatively less activity during the subjective night.

The center and right column in Fig 1 show the activity of a *per*^01^ and a *pdf*^01^ mutant. The behavior of the flies is synchronized by the light-dark cycle, and the flies are mostly active during the day. The behavior of the *per*^01^ mutant presents a strong contrast with the wild type fly, and the activity is evenly distributed during the day. There is a peak of activity when the lights are turned off, that most probably corresponds to a startle response, since no anticipation is observed. The *pdf*^01^ mutant presents both a morning and evening peak in *LD*. As soon as the lights are turned out for the *DD* cycles these peaks disappear and the mutant flies become arrhythmic.

The periodograms calculated from these time series confirm that the period of the wild type flies is 24 hours in *LD* and very close to 24 hs in *DD*, whereas for the clock mutant no peak of the periodogram is above the significance line (see S1 Fig). Thus, when using our custom built arena we recover the well known results about rhythmicity obtained in the standard setups, where the space available to the flies is much more restricted.

Given the high spatial resolution obtained with video tracking, slight movements of the fly, such as grooming, may result in changes in the registered position. Thus, in order to asses the actual displacement of the flies we set a fixed threshold equivalent to the size of a fly (≈ 5 pixels) for the difference in position in consecutive registers, and considered that differences below this threshold correspond to a quiescent fly. If this threshold is slightly increased no qualitative changes are observed in the results. If movement is represented by 1 and rest by 0, now the original time series can be converted to a binary time series. An activity *event* is thus defined as a sequence of consecutive 1’s, whereas a quiescence event is defined as a sequence of 0’s. The *interevent time* is defined as the time elapsed between the beginning of two consecutive activity events.

**Fig 2:**
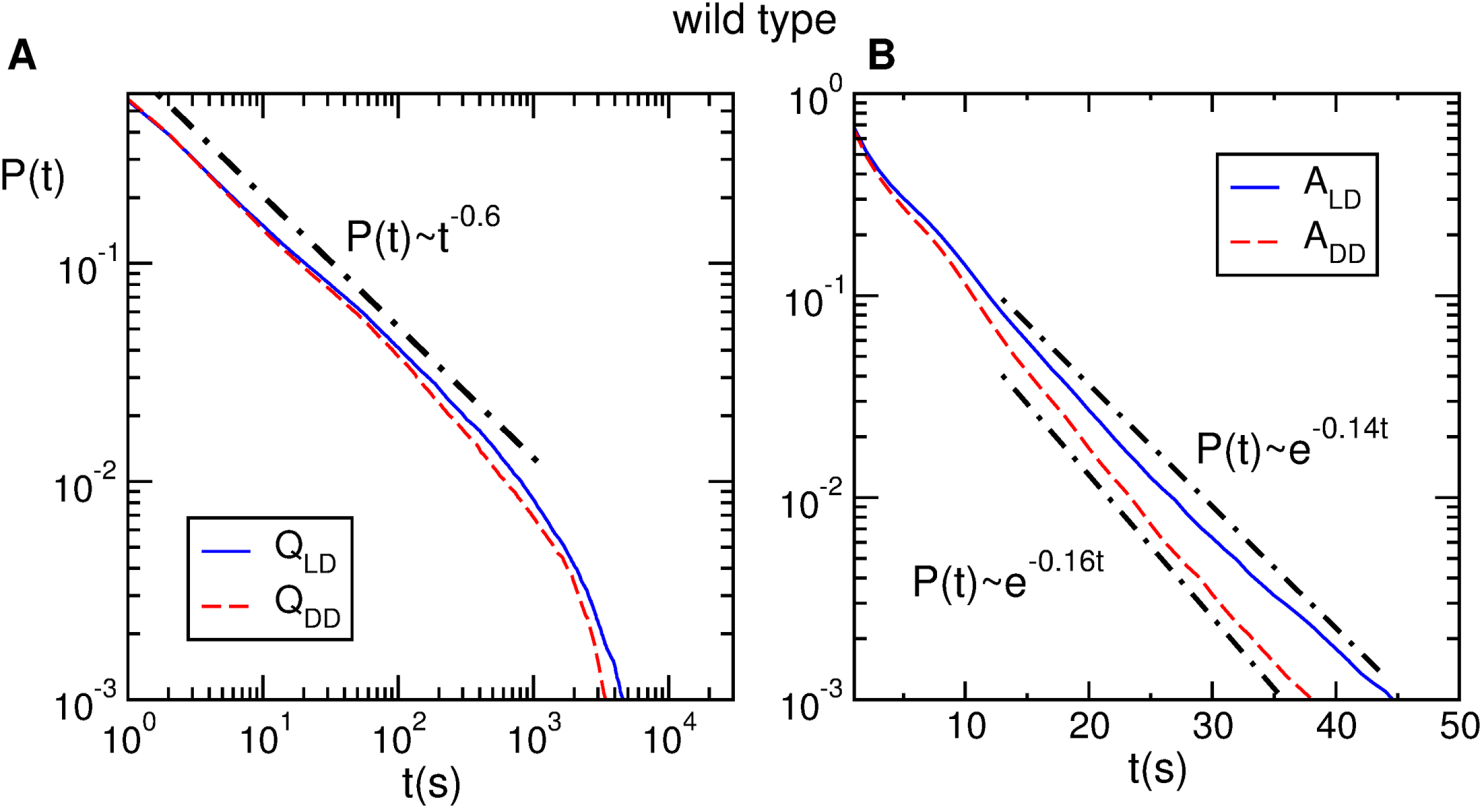
Activity and quiescence interval distributions for wild type. A) Quiescence interval distributions in *LD* and *DD* (*Q*_*LD*_ (continuous) and *Q*_*DD*_ (dashed), respectively) (*n* = 10). B) Activity interval distributions in *LD* and *DD* (*A*_*LD*_ (continuous) and *A*_*DD*_ (dashed), respectively) (*n* = 10). The black dash-dotted lines represent best fits: a power law function in panel A, and exponential functions in panel B. The fitted curves were displaced to avoid superposition with experimental curves.

Fig 2 shows the distributions of quiescence and activity bouts for wild type flies. We have analyzed separately the time series corresponding to *LD* and *DD*. In *LD* we find that the distributions of quiescence and activity can be well fitted by a power law (*p*(*t*) ≈ *t*^−0.6^) and by an exponential distribution (*p*(*t*) ≈ *e*^−0.14*t*^), respectively, as was recently reported both for mice [30] and flies [28].

Also, Fig 2 shows that the exponent of the power law distribution does not change when flies are exposed to *DD* conditions, and the circadian clock is free running. Remarkably, we obtain for the power law almost the same exponent (*α* = 0.6) as that found for *w*^1118^ flies [28], even though the flies we use are of the *CantonS* type. In the case of activity the distribution are well fitted by exponential distributions both in *LD* and *DD* and only a slight change in the exponent was observed (0.14 in *LD* and 0.16 in *DD*. Similar features were also observed in *yw* flies (S3 Fig).

**Fig 3:**
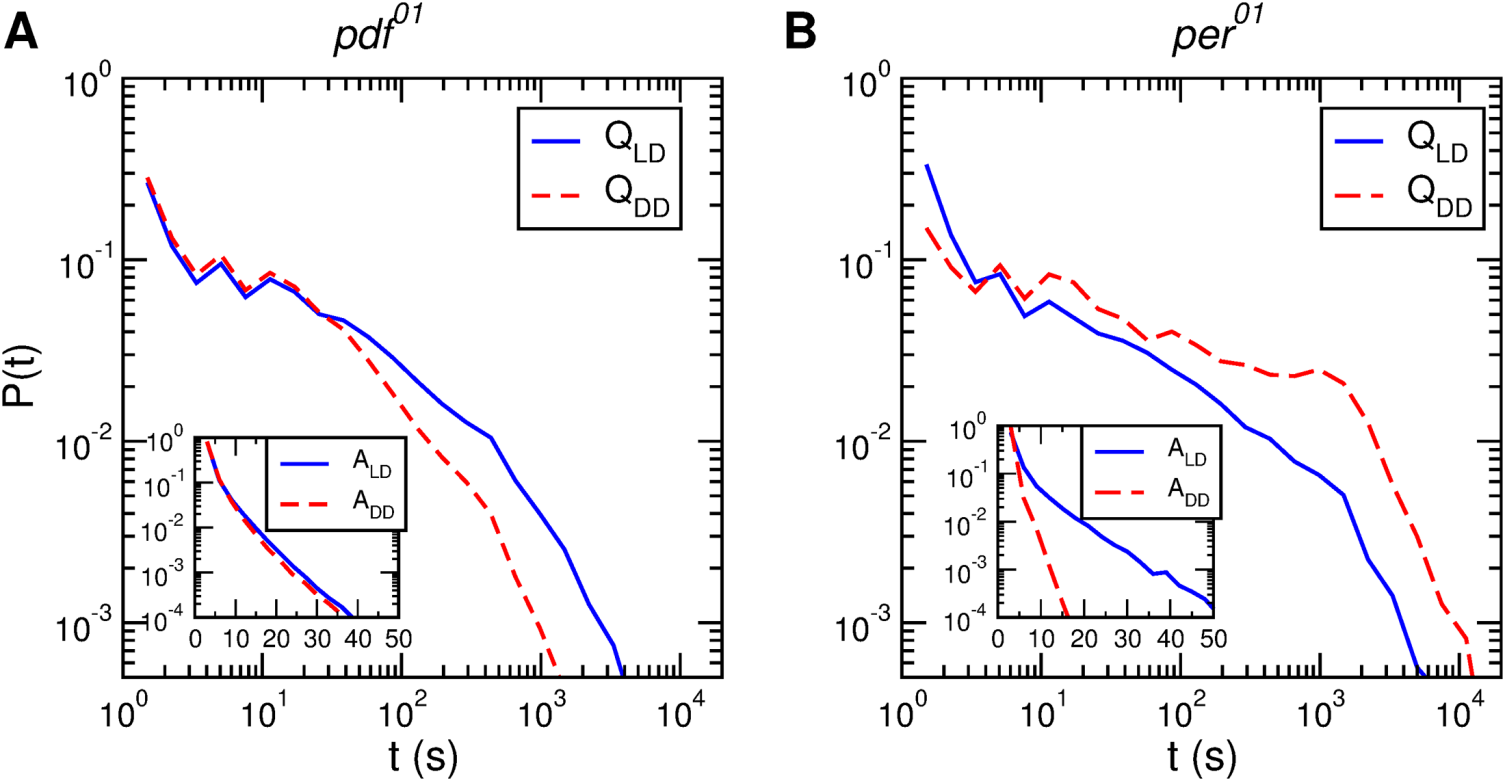
Activity and quiescence distributions for *pdf*^01^ and *per*^01^ mutants. Quiescence distributions in *LD* and *DD* (*Q*_*LD*_ and *Q*_*DD*_, respectively) for (A) *pdf*^01^ (*n* = 17) and (B) *per*^01^ (*n* = 11) mutants. The insets show the respective activity distributions for *LD* and *DD* (*A*_*LD*_ and *A*_*DD*_, respectively). The continuous (dashed) lines present the distributions in *LD* (*DD*).

On the other hand, for both clock mutants, *pdf*^01^ and *per*^01^, we find significant differences between the distributions in *LD* and *DD*. Fig 3 shows that for *LD* the distributions are very broad, and very similar to its counterpart in the wild type. In contrast, in *DD* the shape of the distributions is different in each case. For the wild type, the distributions in *LD* and *DD* are identical in the whole time range. For the *pdf*^01^ mutant these distributions begin to separate at *t* 30*secs*, while for the *per*^01^ the distributions are different in the whole time range. Interestingly, for the *pdf*^01^ mutant the distributions of activity are almost identical. Combined with the fact that the quiescence distribution is different, this means that the flies perform more or less the same activities but have different different rest periods between activity bouts. More specifically, the rest periods are much shorter than in *LD*. This could mean that the neuropeptide pdf is less involved in regulating activity than in regulating rest periods, and that the clock still manages to control activity bouts. In contrast, for *per*^01^ both activity and quiescence distributions are very different, which is probably a signal that the clock is completely disrupted.

**Fig 4:**
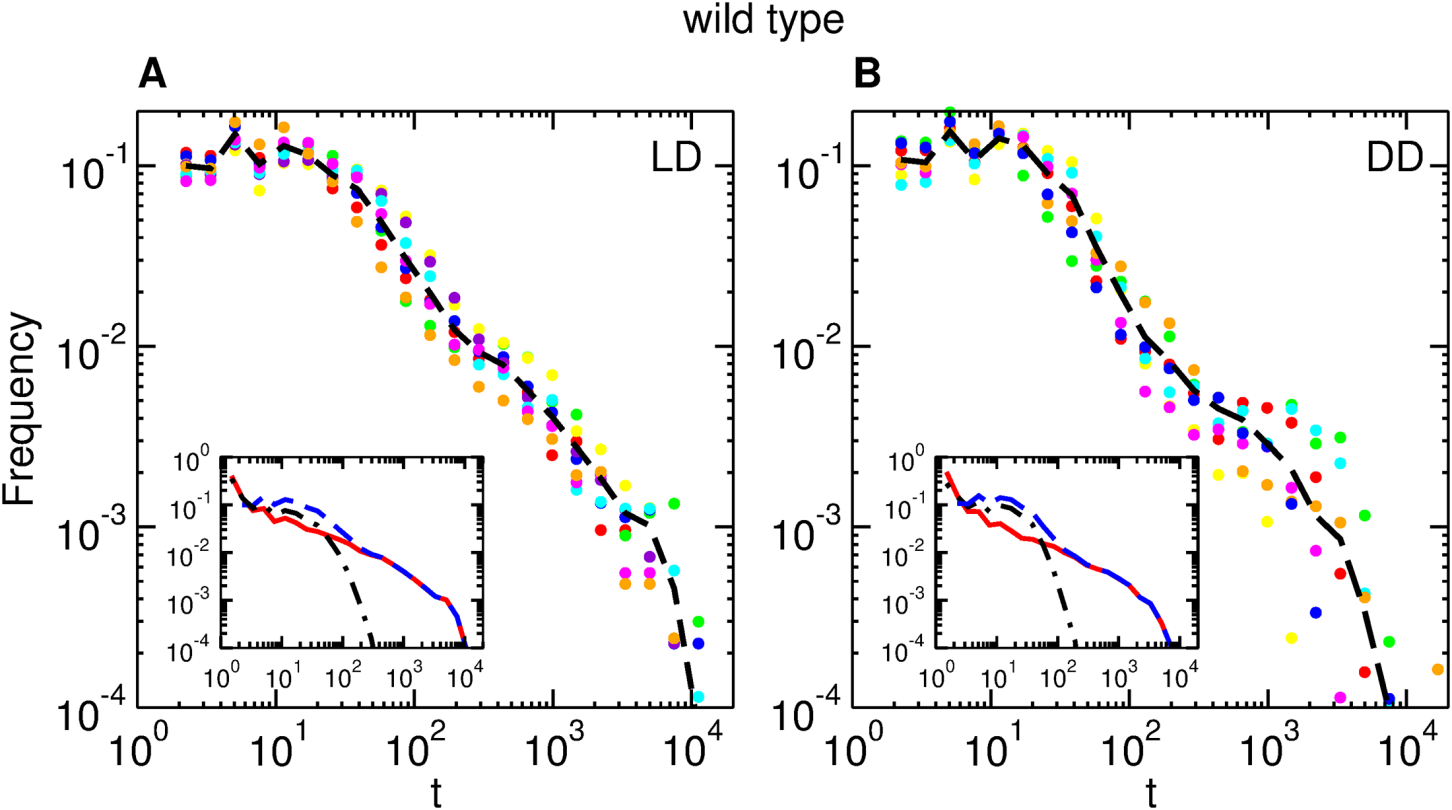
Interevent time distributions for wild type in *LD* and *DD*. The circles of each color represent interevent distributions for single flies (*n* = 10). The dashed line is the average interevent distribution. (A) *LD*, (B) *DD*. Insets: Average distributions of interevent times (dashed blue line), activity bouts (dashed-dotted black line), and quiescence bouts (full, red line).

We turn now to the analysis of interevent times. Fig 4 shows the interevent distributions in *LD* and *DD* for ten different wild type flies, as well as the average interevent distribution. Note that the distributions for all the flies present the same qualitative behavior with a small dispersion. Also, the average distributions present the same qualitative shape for *LD* and *DD*. The insets compare the movement and quiescence distributions with the interevent distribution. Since interevent times are defined as the time elapsed between the onset of two activity bouts, its distribution must be closely related to the distribution of activity and quiescence bouts. In particular, the insets show that, for short times (≲ 200 seconds), the contribution of activity bouts is dominant, whereas for longer times it is the contribution of quiescence bouts which is dominant. In other words, activity bouts tend to be separated by short quiescence bouts, whereas long quiescence bouts tend to be separated by short activity bouts. Interestingly, the same quantitative effect has been observed in Wistar rats [31]. Similar features were also observed in *yw* flies (S4 Fig).

**Fig 5:**
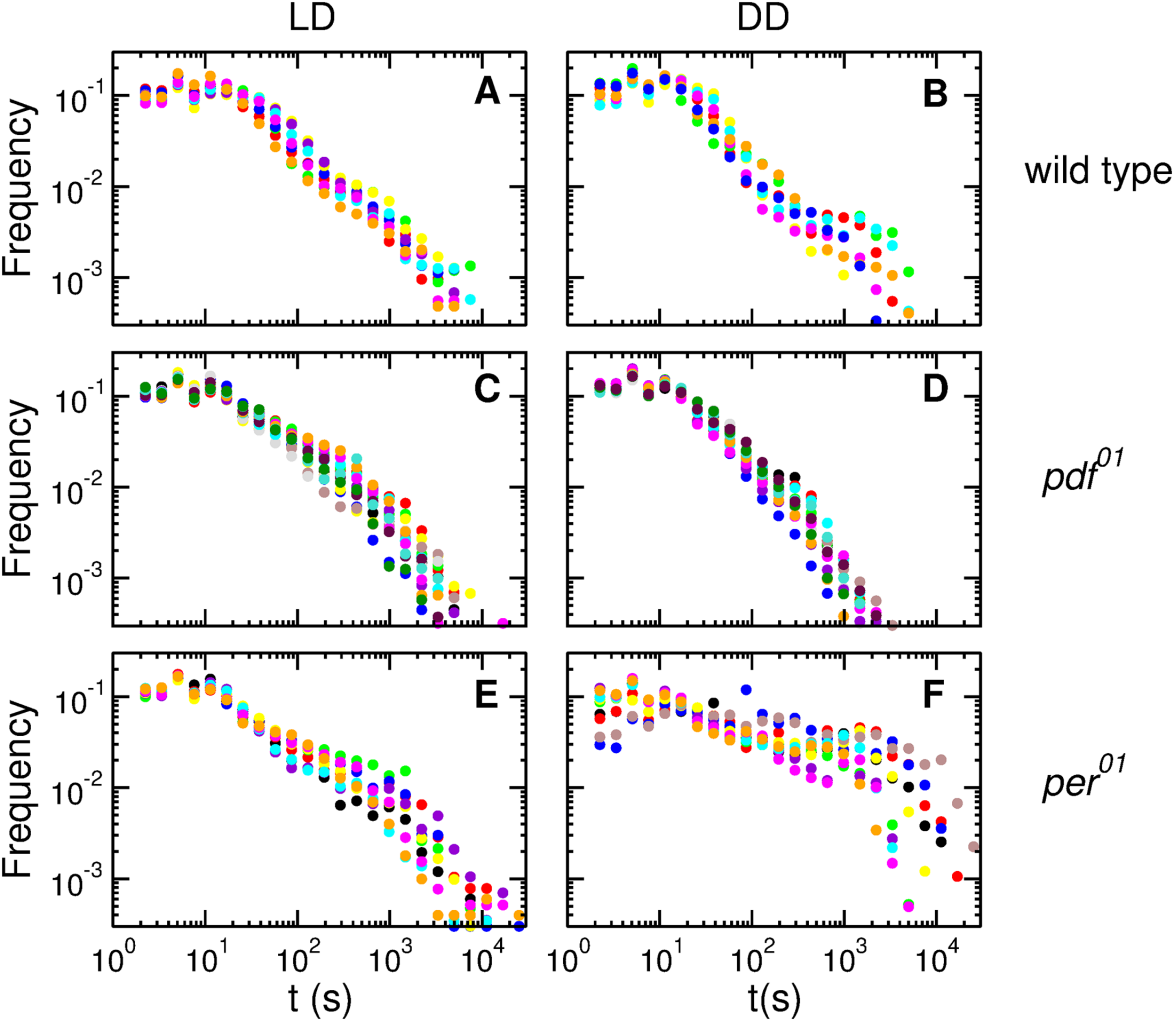
Interevent time distributions for wild type and clock mutants flies in *LD* and *DD*. The circles of each color represent interevent distributions for single flies. (A) Wild type in *LD* (*n* = 10). (B) Wild type in *DD* (*n* = 10). (C) *pdf*^01^ in *LD* (*n* = 17). (D) *pdf*^01^ in *DD* (*n* = 17). (E) *per*^01^ in *LD* (*n* = 11). (F) *per*^01^ in *DD* (*n* = 11).

Fig 5 shows the interevent distributions in *LD* and *DD* for several individual flies of all the genotypes studied. Note first that, just as in the wild type, for the *pdf*^01^ mutant the distributions for all the flies present a small dispersion. However, for *per*^01^ the difference between distributions for individual flies can be rather large. We have also observed these individual differences in a second experiment (see S5 Fig). As happens with the distributions of activity and quiescence intervals, in *LD* the interevent distributions of the wild type and the two clock mutants are qualitatively similar, displaying a slow decay and a cutoff. Again, for the clock mutants there are important differences in the distributions between LD and DD. For the *pdf*^01^ the distribution in *DD* becomes less broad (for all individual flies). For *per*^01^ the dispersion of the individual distributions in *DD* becomes much larger (note that Fig 5 is in log-log scale).

**Fig 6:**
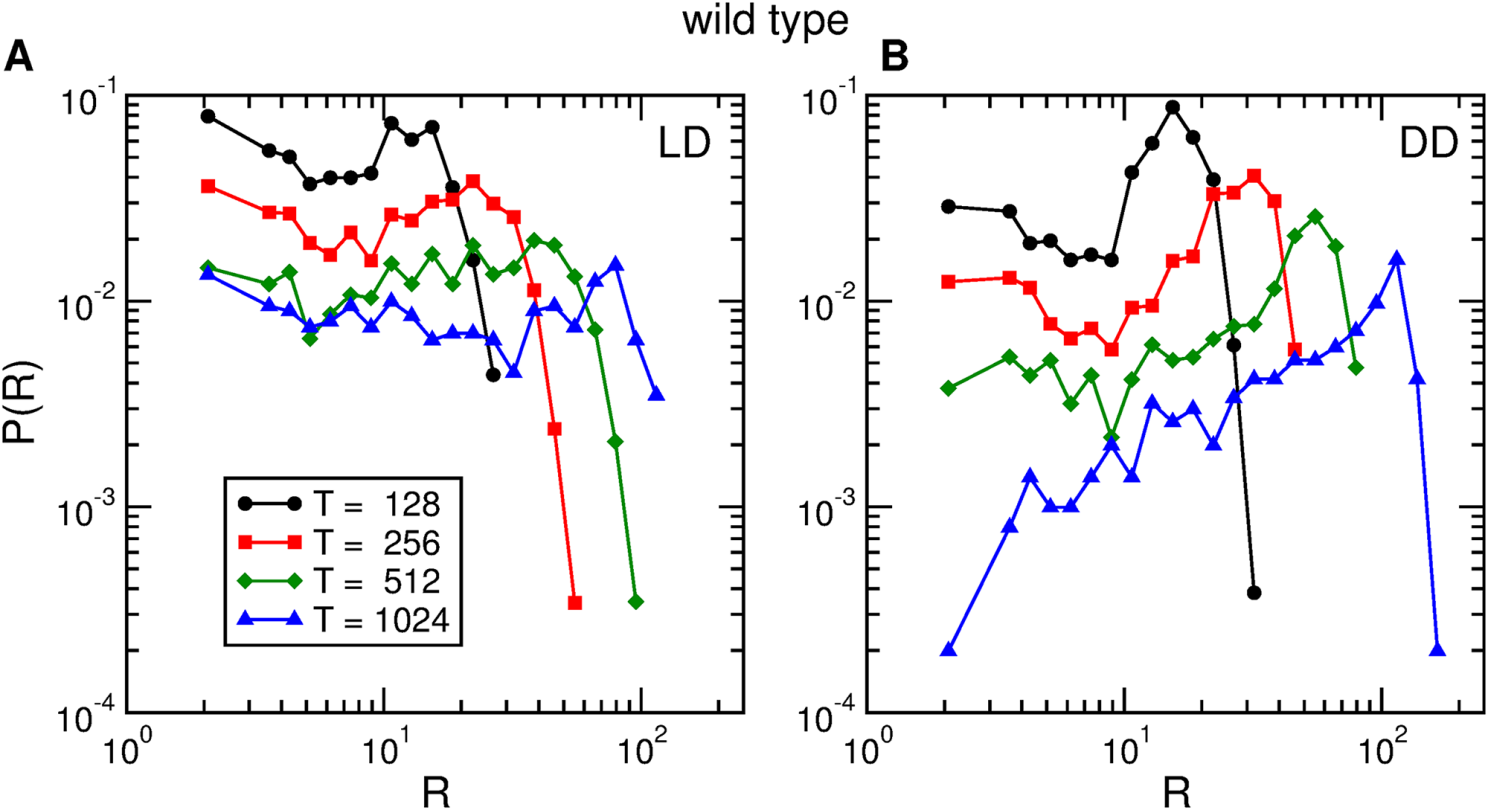
Activity rate distributions. Distribution of the rates of activity events of one wild type fruit fly, in (A) *LD* conditions and (B) *DD* conditions, for four time windows of widths *T* = 128, 256, 512, and 1024 seconds.

A different, but complementary, characterization of animal movement can be obtained if we switch our attention from the *duration* of activity bouts, to the *timing* of such bouts. In particular, we have focused our attention in the *rate* *R* of activity bouts [31], defined as the number of bouts that are contained in time windows of a given length *T*. It is evident that these rates depend of the moment when each time window is sampled. In particular, the clear difference in activity levels between day and night (see Fig 1) implies that event rates during the day are very different from event rates during the night. But the time dependence of local rates runs much deeper than this: Fig 6 shows that when the fly is in LD conditions, the distributions are very broad followed by an exponential decay. The robustness of this result is confirmed by the fact that very similar distributions are obtained when other widths for the time windows are considered (see Fig 6). Interestingly, it has been reported [31] that, in Wistar rats, rate distributions also have very long tails. Note that, both in *LD* and *DD* as the time window is increased the distributions are shifted, but otherwise maintain their functional form.

**Fig 7:**
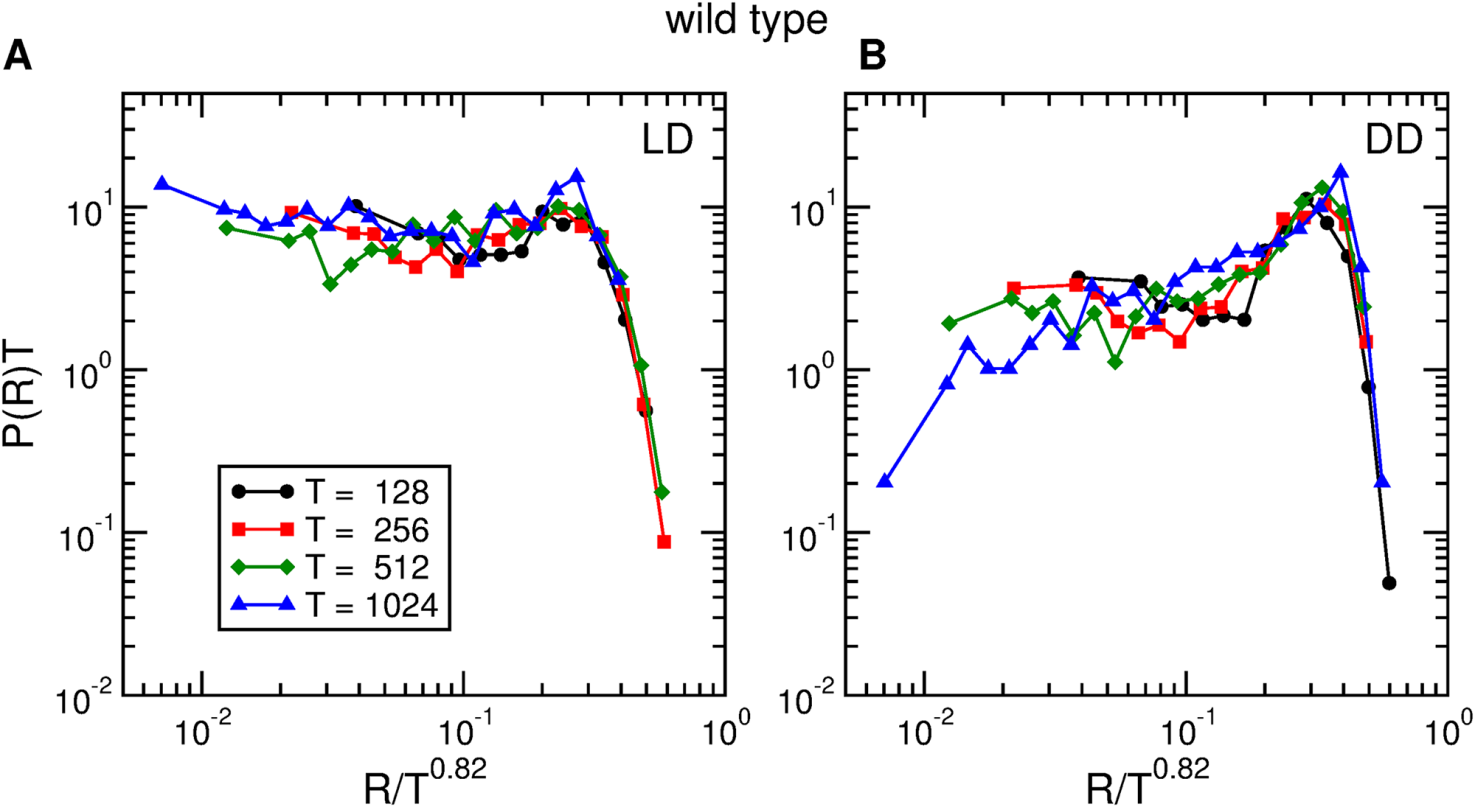
Activity rate scaling for wild type. Scaled distributions of activity rates of one wild type fruit fly, in (A) *LD* conditions and (B) *DD* conditions, for four time windows of widths *T* = 128, 256, 512, and 1024 seconds.

The differences in the rate distributions when different time windows are considered can be explained as a consequence of finite size scaling [32]. In Fig 7A we rescale all the distributions of rates presented in Fig 6 as *P*(*R*)*T* vs. *R*/*T*^0.82^. The figure shows that the collapse obtained is reasonably good. Using the same scaling function we also obtain a good collapse for the rate distributions in DD conditions (see Fig 7B). Even though the results shown here are for one individual wild type fly, S6 Fig shows that the same scaling function gives reasonably good collapses for the rate distributions of all the wild type flies used in the experiment.

**Fig 8:**
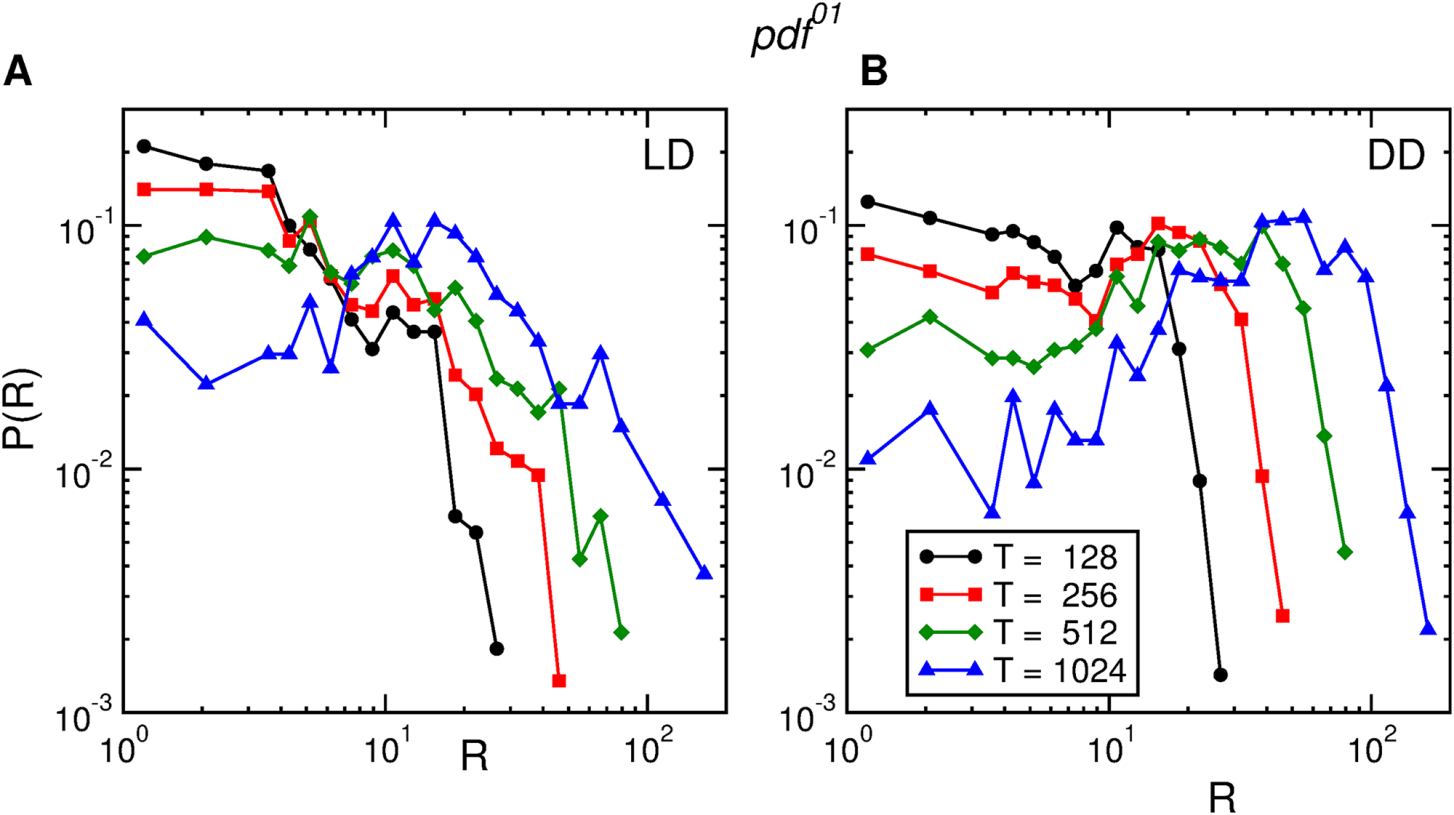
Activity rate distributions for *pdf*^01^. Distribution of rate of activity events of one *pdf*^01^ fruit fly, in *LD* conditions (A), and in *DD* conditions (B), for four time windows of widths *T* = 128, 256, 512, and 1024 seconds.

**Fig 9:**
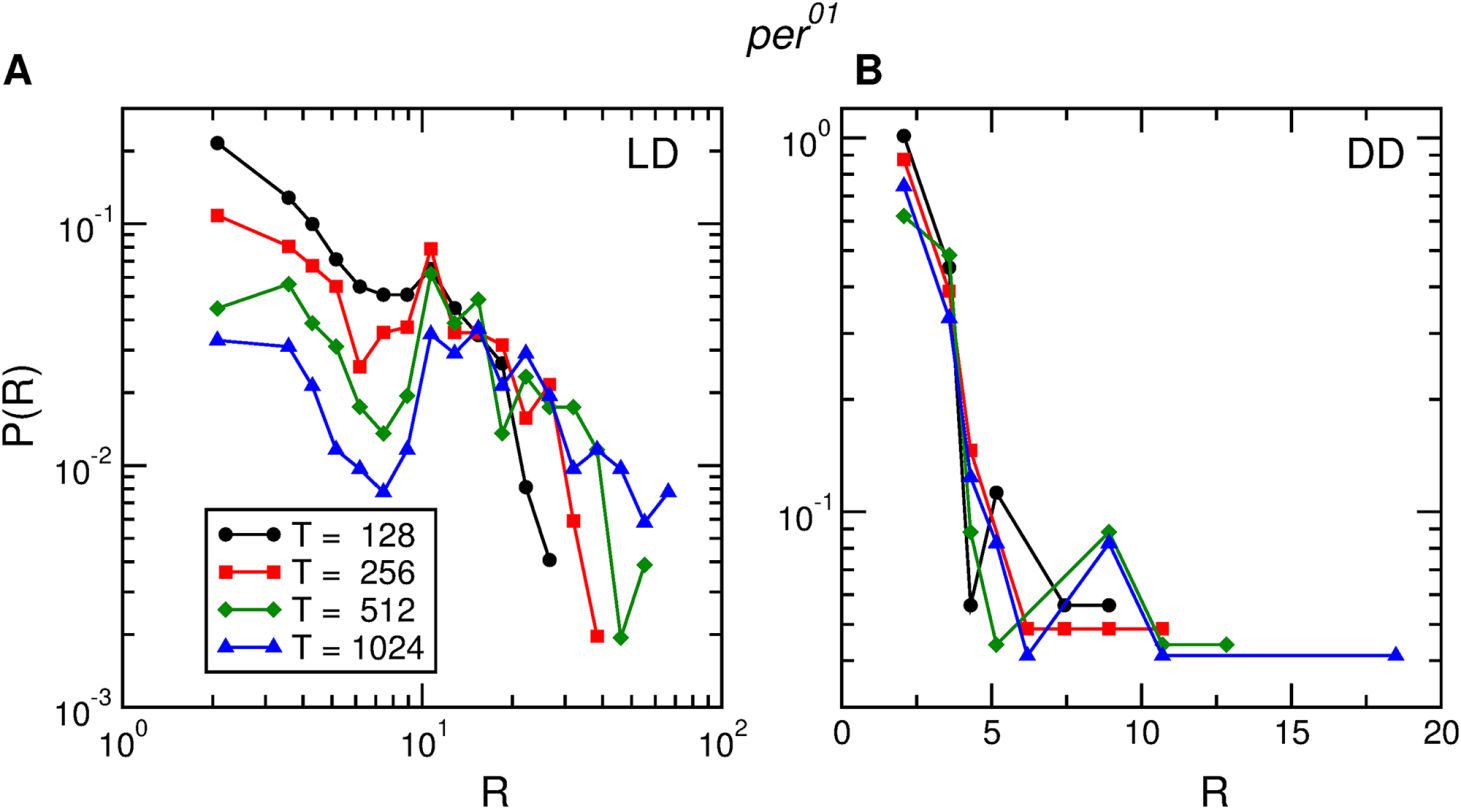
Activity rate distributions for *per*^01^. Distribution of rate of activity events for one *per*^01^ fruit fly, in (A) *LD* conditions and (B) *DD* conditions, for four time windows of widths *T* = 128, 256, 512, and 1024 seconds.

Both for the *pdf*^01^ (Fig 8) and *per*^01^ (Fig 9) clock mutants the activity rate distributions cannot be rescaled neither in *LD* nor in *DD*. S7 and S8 Figs show the same qualitative behavior for all the other *pdf*^01^ and *per*^01^ flies used in the experiment.

## Discussion

It is well known that the circadian clock interacts with the system generating locomotor activity in *Drosophila melanogaster* and in many other species. However, until very recently, only the most basic features of locomotor activity were studied. In order to get a more detailed picture of its interaction with the circadian clock, we have analyzed some statistical properties of the locomotor activity of both wild type flies and two different clock mutants, *per*^01^ and *pdf*^01^. The reasons why the endogenous circadian clock is not functional are not the same for each mutant. *per*^01^ flies lack the protein PER [33], which is necessary to have working cellular clocks. Whereas in *pdf*^01^ mutants it is the neuropeptide PDF (pigment dispersing factor) that is lacking [34]. This neuropeptide has been shown to be essential for the communication between cellular clocks. Thus, in *pdf*^01^ the cellular clocks are in principle intact, but they cannot generate a coherent behavioral rhythm [35].

In principle, differences in the statistical properties of the activity of wild type and clock mutants could be due to motor deficiencies besides those in the circadian mechanism. However, this does not seem to be the case, since the qualitative behaviors we observed in *LD* are similar in all the genotypes analyzed, while striking differences were observed in *DD*. In *LD* light acts as a temporal references, that can be used by all lines, which is probably the reason why all genotypes were entrained in *LD*. In *DD* this reference is lost, and only a working endogenous clock can provide this reference. For the wild type, the distribution of activity and quiescence bouts in LD conditions can be well approximated by exponential and power law distributions, respectively, which is in agreement with previous results for Drosophila [27, 28] and Wistar rats [31]. Interestingly, the power law function that fits our quiescence data has the same exponent as obtained by Ueno et al. [28]. They monitored the activity of fruit flies with a different genotype, and in arenas rather different (circular, with a diameter of 5 cm.) from the one we have used. This supports the idea that these behaviors are robust, and in fact it has been argued [36] that they may be present in many species, even when the possibility of flying is taken into account. In DD we found that the quiescence and activity bouts of the wild type have the same distribution as in LD. In contrast, in the clock mutants we found that there is a significant difference between the distributions in LD and DD. This shows that, even though it is well known that the circadian clock imposes a circadian rhythmicity in locomotor activity, it is also able to control locomotor activity at time scales that are much shorter than one day.

It is reasonable to expect that the clock may affect the locomotor activity at different time scales. This could be due to a direct effect on all time scales, or also by a hierarchical structure of time scales, that is somehow affected at a given level, and then propagates this perturbation to downstream levels. Our results suggest that this might be the case, since the statistics of movement have important differences in the two clock mutants studied here. We find that in both mutants the distributions of interevent times are different in *LD* and *DD*, but the dispersion in the intervent times for individual flies is very large in *per*^01^, whereas it is rather small in *pdf*^01^ (almost as small as in the wild type). In the case of the rate of activity bouts calculated using different time windows, we find that the distribution display finite size scaling in LD and DD for the wild type, but they cannot be rescaled in the *pdf*^01^ and *per*^01^ mutants. Interestingly, the finite size scaling function we find is the same for all the wild type flies, and is very similar to the one found for Wistar rats [31].

Our results suggest that the circadian clock influences locomotor activity at time scales that can be much shorter than one day. Considering that the circadian network in the brain of *Drosophila melanogaster* consists of a few neuronal groups which have very different roles in the control of various behavioral traits, it would be interesting to study the statistics of movement in other clock mutants, in order to see if the control of different statistics is related to different neuronal groups. Also, given the similarities between some of our results and their counterparts in mammals, it would be interesting to see whether in mammals there are also differences between clock mutants for any aspects of locomotor activity.

## Supporting information

S1 Fig Periodograms for wild type and *per*^01^ mutant. The dashed line represents the significance level (*p* = 0.05). Wild type flies have a rhythm of 24.04hs in *DD*, while the *per*^01^ mutants are arrhythmic.
(PDF)

S2 Fig Average activity plots for wild type, *per*^01^ and *pdf*^01^ mutants. Average distance as a function of time presented as double plotted activity plots for wild type (left), *per*^01^ (center), and *pdf*^01^ (right). The plots show both *LD* and *DD* conditions.
(PDF)

S3 Fig Activity and quiescence distributions for *yw* flies. (A) Quiescence distributions for *yw* flies in *LD* and *DD* (*Q*_*LD*_ (continuous) and *Q*_*DD*_ (dashed), respectively) (n = 10). (B) Activity interval distribution for *yw* flies in *LD* and *DD* (*A*_*LD*_ (continuous) and *A*_*DD*_ (dashed), respectively) (n = 10). The black dash-dotted lines represent best fits: a power law function in panel *A*, and exponential functions in panel *B*. The fitted curves were displaced to avoid superposition with experimental curves.
(PDF)

S4 Fig Interevent time distributions for *yw* flies in *LD* and *DD*. The circles of each color represent interevent distributions for single *yw* flies (n = 10). The dashed line is the average interevent distribution in *LD* (left) and *DD* (right). The insets show the average distributions of interevents times (dashed blue line), activity bouts (dashed-dotted black line), and quiescence bouts (full, red line).
(PDF)

S5 Fig Interevent time distributions for two different experiments with *per*^01^ mutant. The top row correspond to the first experiment (*n* = 6), while the second row corresponds to the second experiment (*n* = 6). The figures on the left correspond to *LD* while the figures on the right to *DD* conditions. The circles of each color represent interevent distributions for individual flies.
(PDF)

S6 Fig Activity rate scaling for wild type. Scaled distribution of activity rate for eight wild type fruit flies in *LD* conditions, for four time windows *T* = 128, 256, 512 and 1024 seconds.
(PDF)

S7 Fig Activity rate distribution for *pdf*^01^. Distribution of activity rates for six *pdf*^01^ flies in *LD* conditions (*A*), and *DD* conditions (*C*), for four time windows *T* = 128, 256, 512 and 1024 seconds.
(PDF)

S8 Fig Activity rate distribution for *per*^01^. Distribution of activity rates for ten *per*^01^ flies in *LD* conditions (left column), and *DD* conditions (right column), for four time windows *T* = 128, 256, 512 and 1024 seconds.
(PDF)
