# Supplementary material for: Role of the circadian clock in the statistics of locomotor activity in Drosophila
Source: PLoS One. 2018 Aug 23;13(8):e0202505. doi: 10.1371/journal.pone.0202505 (PMC6107170; doi:10.1371/journal.pone.0202505)
Supplement: S1 Fig — The dashed line represents the significance level (p = 0.05). Wild type flies have a rhythm of 24.04hs in DD, while the per01 mutants are arrhythmic. (PDF) [file pone.0202505.s001.pdf]

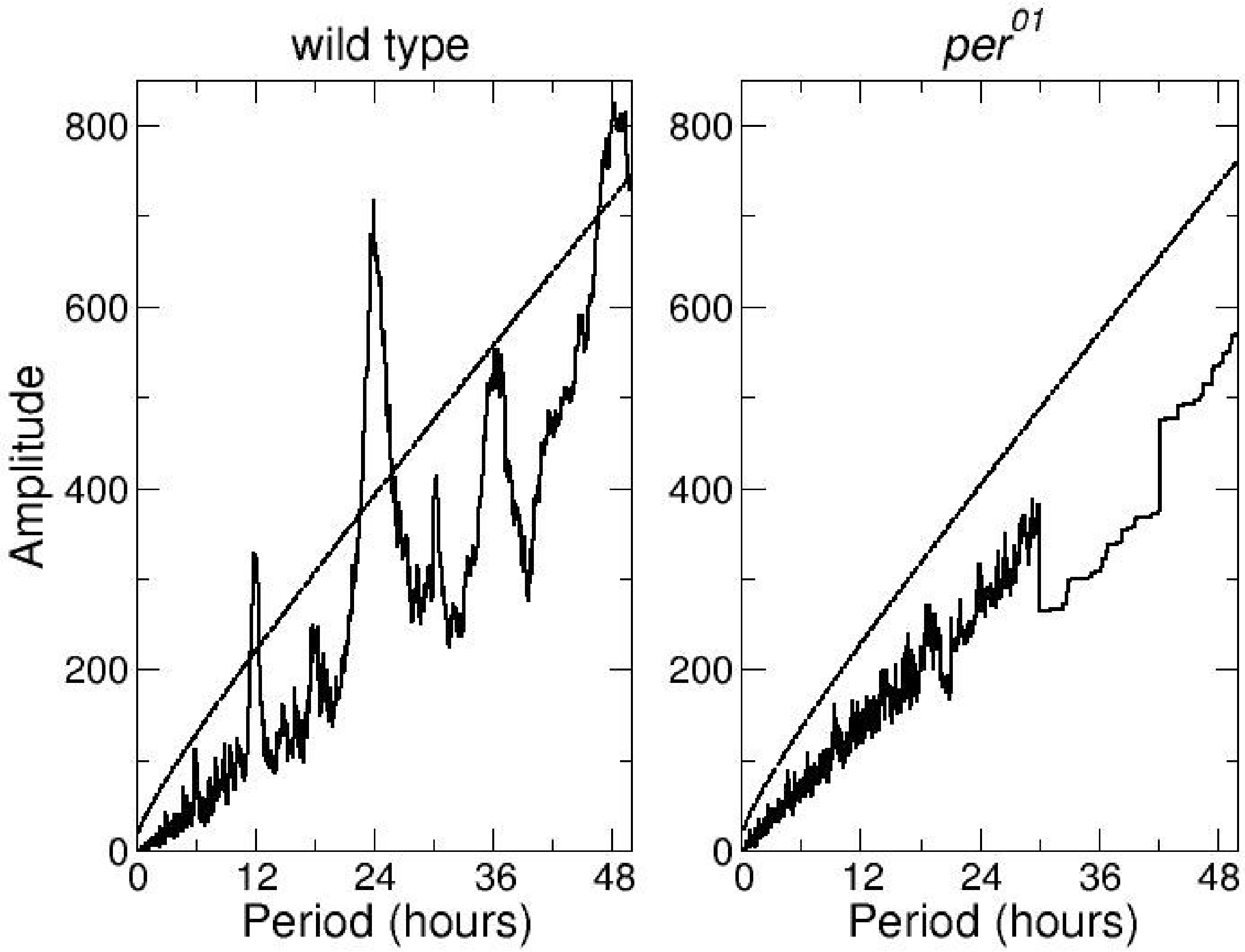

Figure S1: Periodograms for wild type and *per*^{01} mutant.
The dashed line represents the significance level (p=0.05). Wild type flies have a rhythm of 24.04hs in DD, while the *per*^{01} mutants are arrhythmic.
