# Supplementary material for: Role of the circadian clock in the statistics of locomotor activity in Drosophila
Source: PLoS One. 2018 Aug 23;13(8):e0202505. doi: 10.1371/journal.pone.0202505 (PMC6107170; doi:10.1371/journal.pone.0202505)
Supplement: S2 Fig — Average distance as a function of time presented as double plotted activity plots for wild type (left), per01 (center), and pdf01 (right). The plots show both LD and DD conditions. (PDF) [file pone.0202505.s002.pdf]

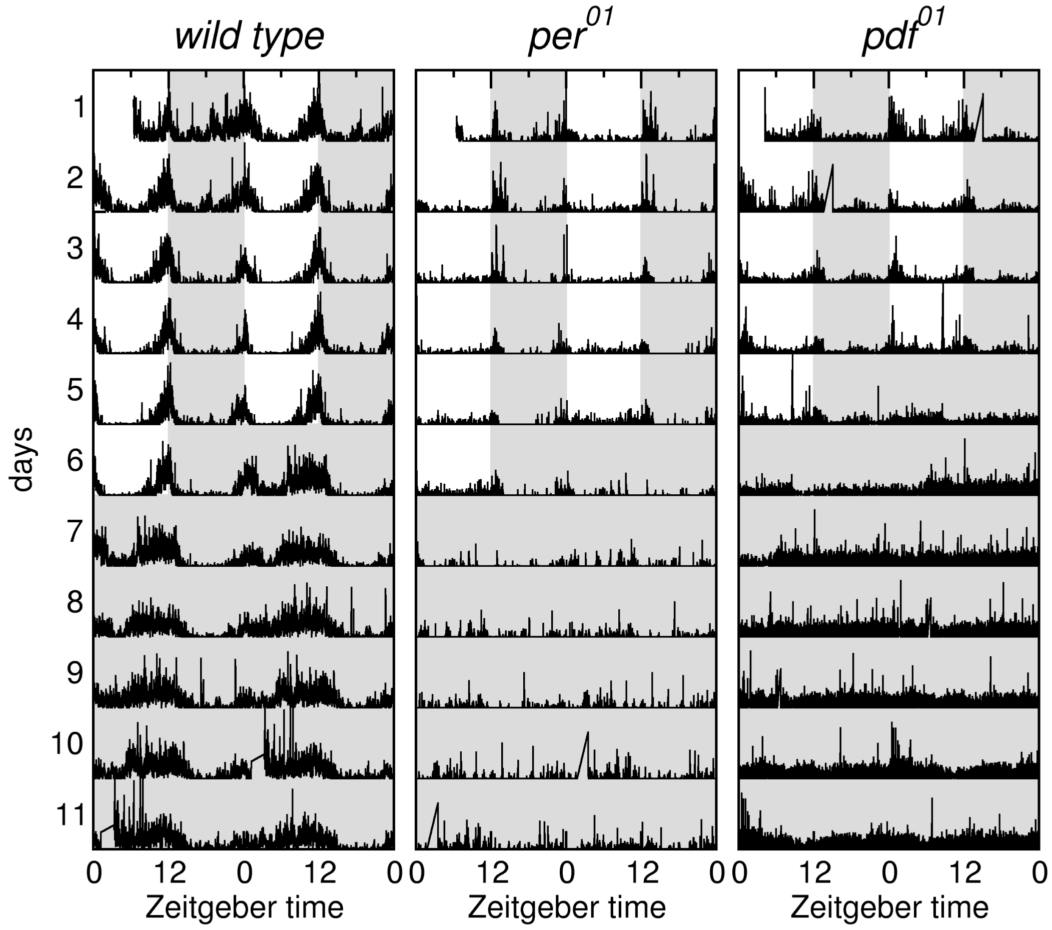


**Figure S2: Average activity plots for wild type, *per*$^{01}$ and *pdf*$^{01}$ mutants.**
Average distance as a function of time presented as double plotted activity plots for *wild type* (left), *per*$^{01}$ (center), and *pdf*$^{01}$ (right). The plots show both LD and DD conditions.
