# Supplementary material for: Role of the circadian clock in the statistics of locomotor activity in Drosophila
Source: PLoS One. 2018 Aug 23;13(8):e0202505. doi: 10.1371/journal.pone.0202505 (PMC6107170; doi:10.1371/journal.pone.0202505)
Supplement: S3 Fig — (A) Quiescence distributions for yw flies in LD and DD (QLD (continuous) and QDD (dashed), respectively) (n = 10). (B) Activity interval distribution for yw flies in LD and DD (ALD (continuous) and ADD (dashed), respectively) (n = 10). The black dash-dotted lines represent best fits: a power law function in panel A, and exponential functions in panel B. The fitted curves were displaced to avoid superposition with experimental curves. (PDF) [file pone.0202505.s003.pdf]

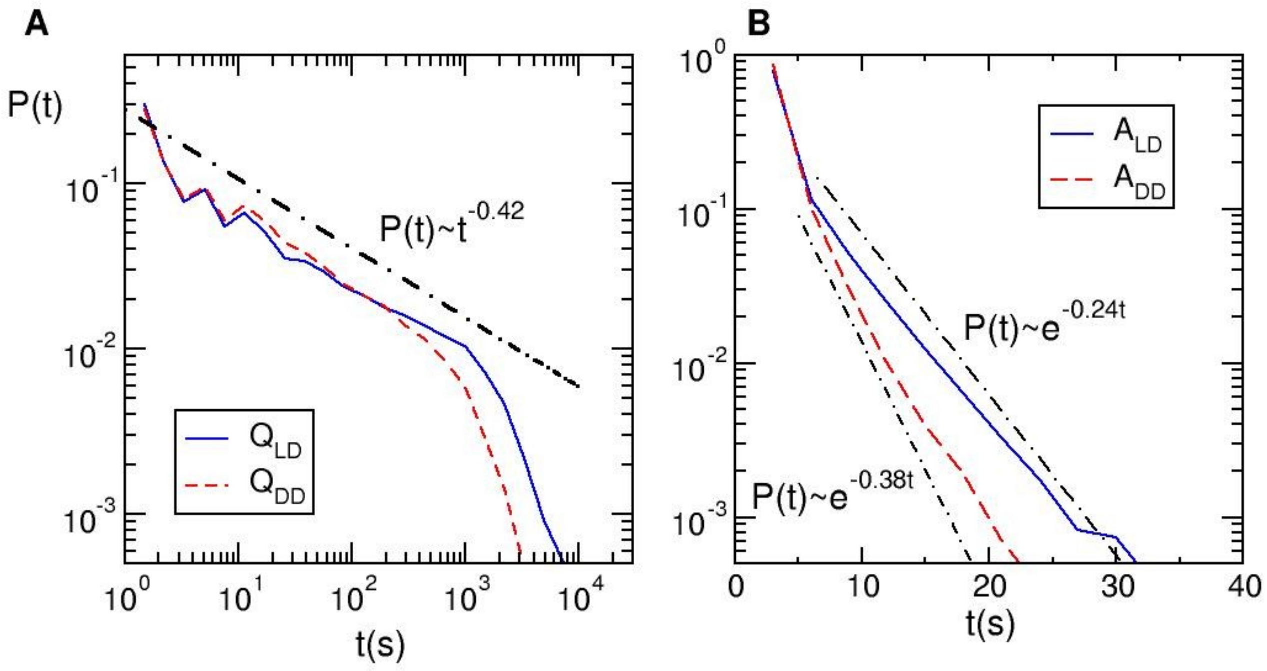

Figure S3: Activity and quiescence distributions for *yw.*

(A) Quiescence distributions for *yw* flies in LD and DD (Q_{LD} (continuous) and Q_{DD} (dashed), respectively) (n=10). (B) Activity interval distribution for *yw* flies in LD and DD (A_{LD} (continuous) and A_{DD} (dashed), respectively) (n=10). The black dash-dotted lines represent best fits: a power law function in panel A, and exponential functions in panel B. The fitted curves were displaced to avoid superposition with experimental curves.
