# Supplementary material for: Role of the circadian clock in the statistics of locomotor activity in Drosophila
Source: PLoS One. 2018 Aug 23;13(8):e0202505. doi: 10.1371/journal.pone.0202505 (PMC6107170; doi:10.1371/journal.pone.0202505)
Supplement: S4 Fig — The circles of each color represent interevent distributions for single yw flies (n = 10). The dashed line is the average interevent distribution in LD (left) and DD (right). The insets show the average distributions of interevents times (dashed blue line), activity bouts (dashed-dotted black line), and quiescence bouts (full, red line). (PDF) [file pone.0202505.s004.pdf]

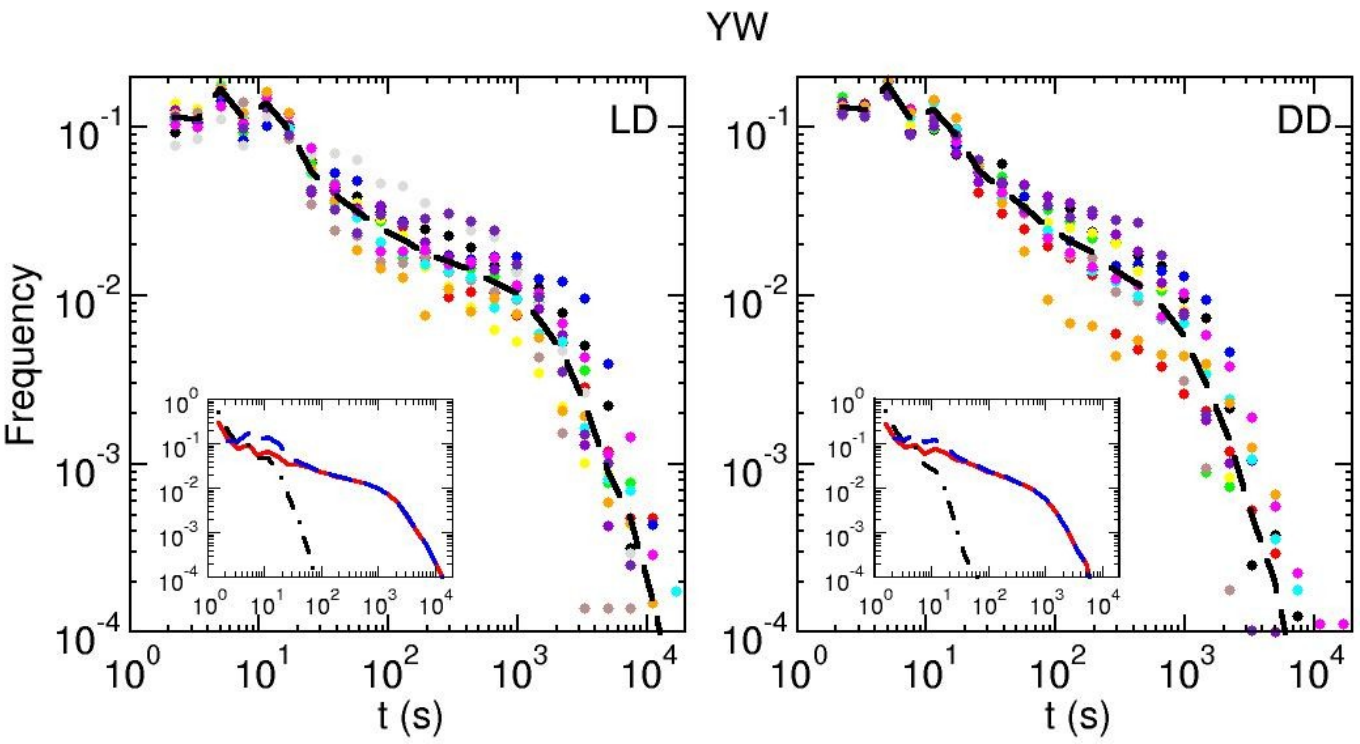

Figure S4: Interevent time distributions for *yw* flies in LD and DD.

The circles of each color represent interevent distributions for single *yw* flies (n=10). The dashed line is the average interevent distribution in LD (left) and DD (right). The insets show the average distributions of interevents times (dashed blue line), activity bouts (dashed-dotted black line), and quiescence bouts (full, red line).
