# Supplementary material for: Role of the circadian clock in the statistics of locomotor activity in Drosophila
Source: PLoS One. 2018 Aug 23;13(8):e0202505. doi: 10.1371/journal.pone.0202505 (PMC6107170; doi:10.1371/journal.pone.0202505)
Supplement: S5 Fig — The top row correspond to the first experiment (n = 6), while the second row corresponds to the second experiment (n = 6). The figures on the left correspond to LD while the figures on the right to DD conditions. The circles of each color represent interevent distributions for individual flies. (PDF) [file pone.0202505.s005.pdf]

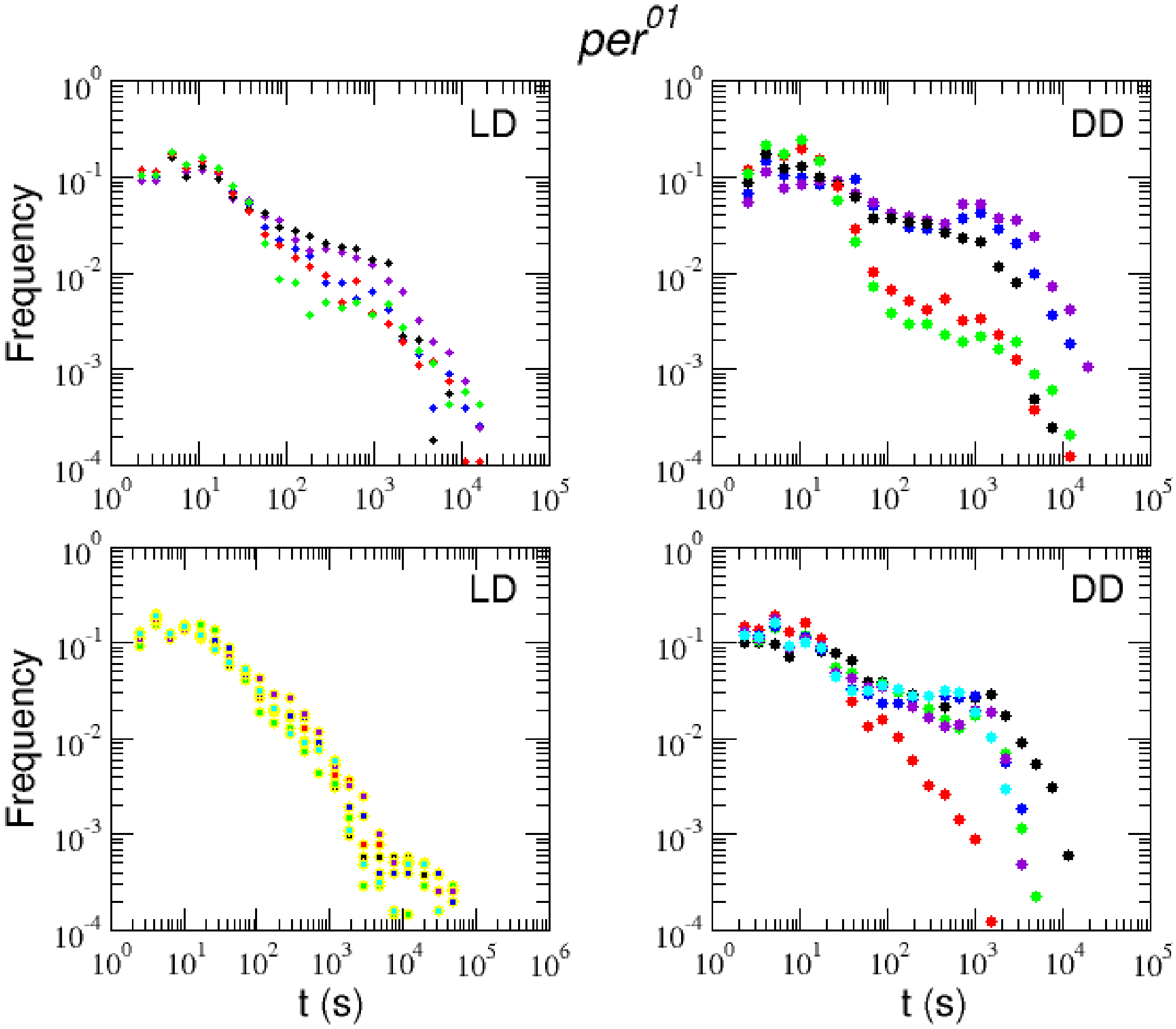

Figure S5: Interevent time distributions for two different experiments with *per*^{01} mutant. The top row correspond to the first experiment (n=6), while the second row corresponds to the second experiment (n=6). The figures on the left correspond to LD while the figures on the right to DD conditions. The circles of each color represent interevent distributions for individual flies.
