# Supplementary material for: Role of the circadian clock in the statistics of locomotor activity in Drosophila
Source: PLoS One. 2018 Aug 23;13(8):e0202505. doi: 10.1371/journal.pone.0202505 (PMC6107170; doi:10.1371/journal.pone.0202505)
Supplement: S6 Fig — Scaled distribution of activity rate for eight wild type fruit flies in LD conditions, for four time windows T = 128, 256, 512 and 1024 seconds. (PDF) [file pone.0202505.s006.pdf]

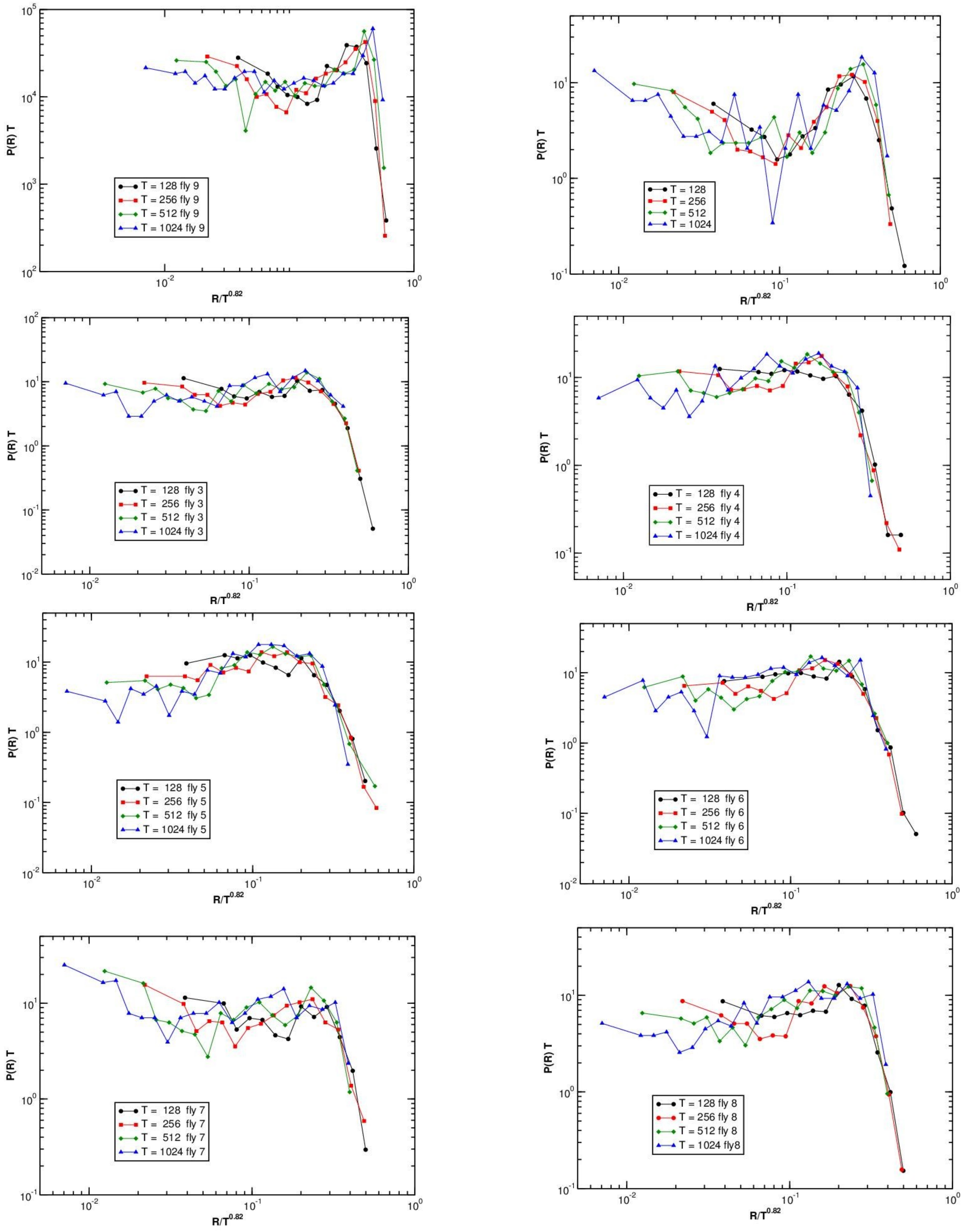

Figure S6: Activity rate scaling for wild type

Scaled distribution of activity rate for eight wild type fruit flies in LD conditions, for four time windows T = 128, 256, 512 and 1024 seconds.
