# Supplementary material for: Role of the circadian clock in the statistics of locomotor activity in Drosophila
Source: PLoS One. 2018 Aug 23;13(8):e0202505. doi: 10.1371/journal.pone.0202505 (PMC6107170; doi:10.1371/journal.pone.0202505)
Supplement: S7 Fig — Distribution of activity rates for six pdf01 flies in LD conditions (A), and DD conditions (C), for four time windows T = 128, 256, 512 and 1024 seconds. (PDF) [file pone.0202505.s007.pdf]

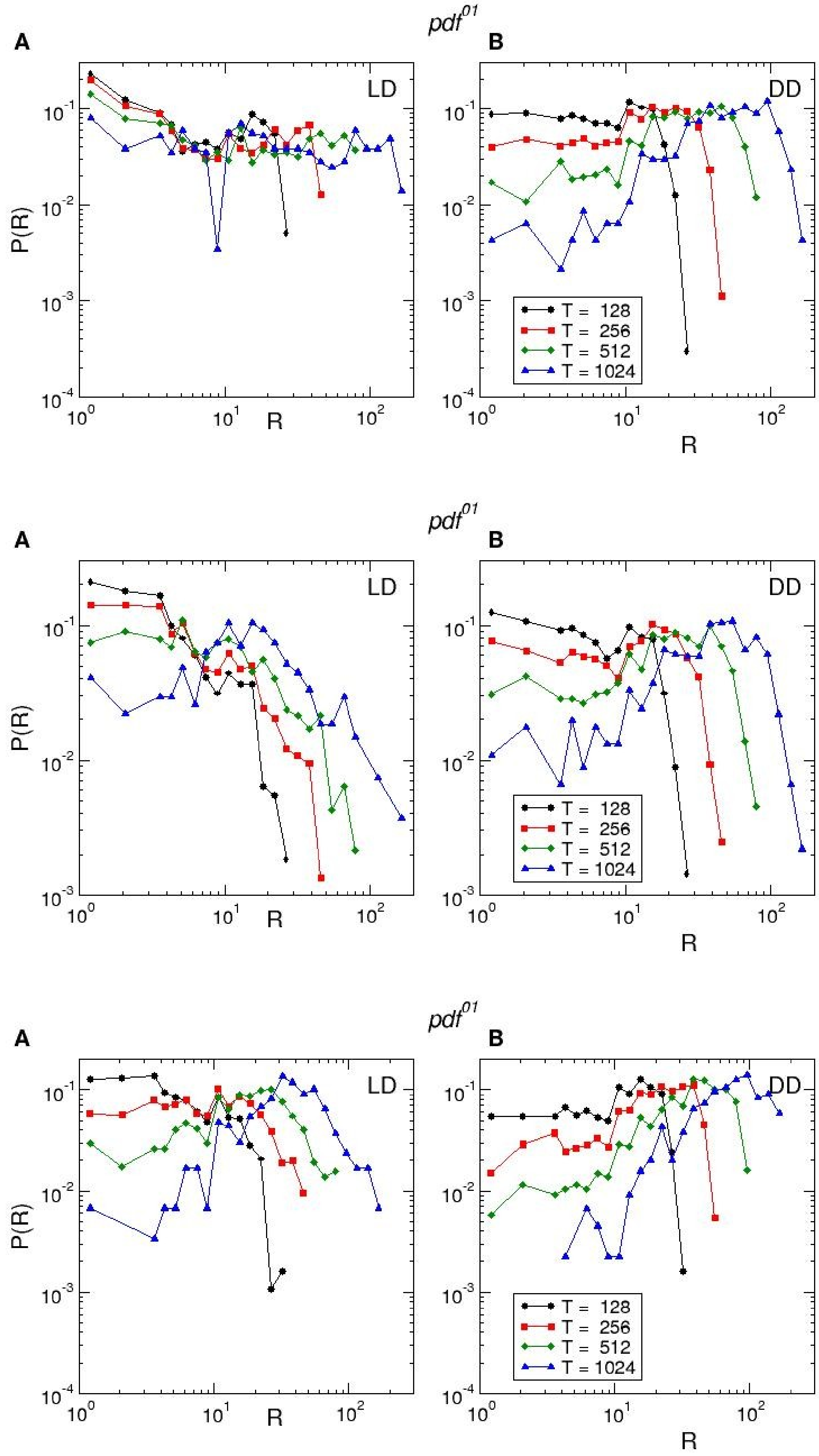

Figure S7: Activity rate distribution for *pdf*[01]
Distribution of activity rates for ten *pdf*[01] flies in LD conditions (A), and DD conditions (B), for four time windows T = 128, 256, 512 and 1024 seconds.

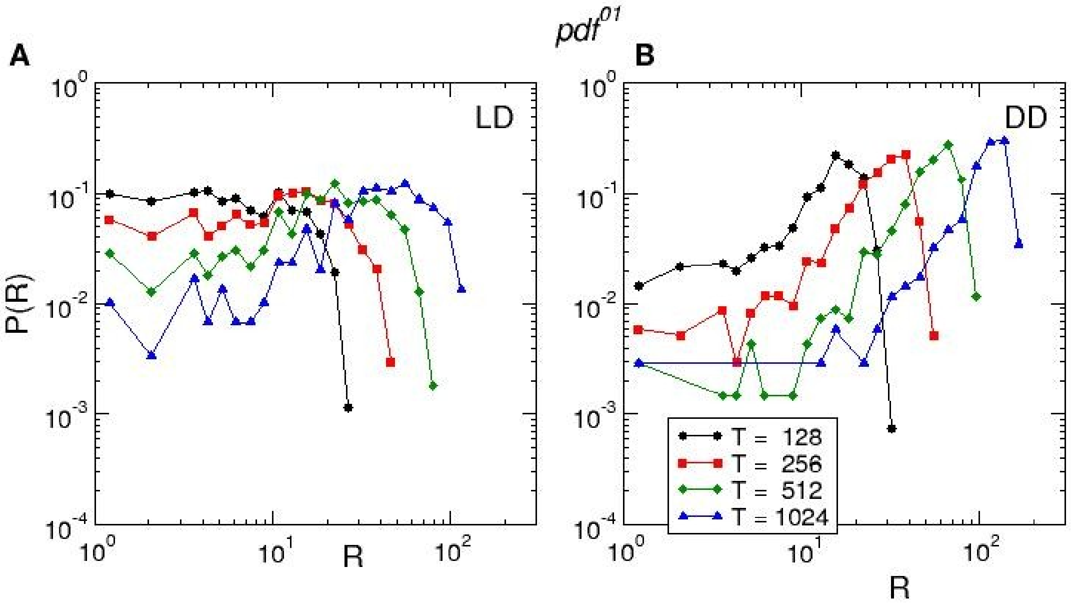

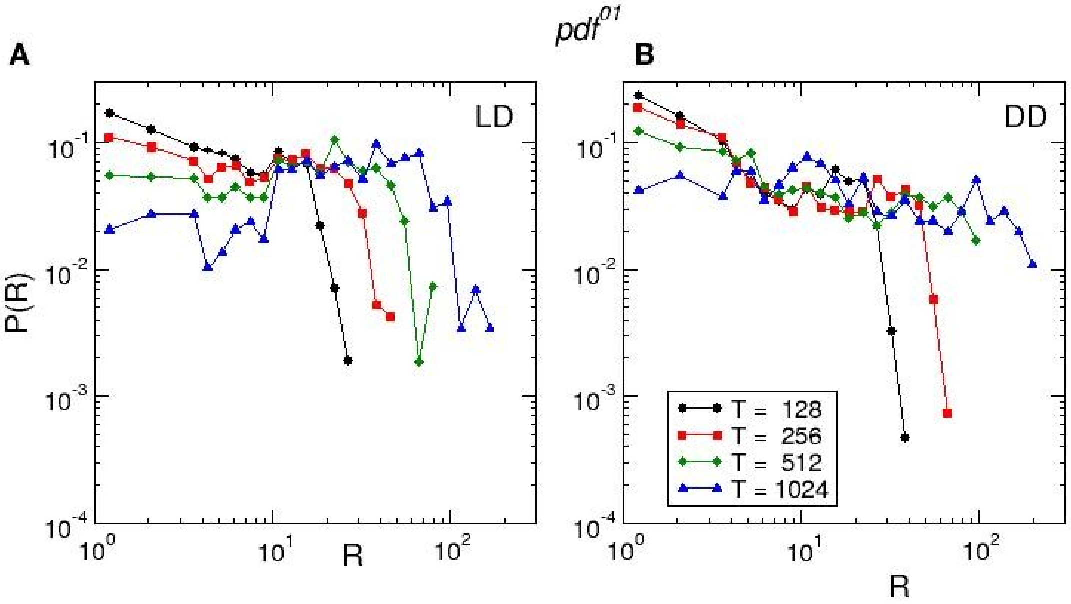

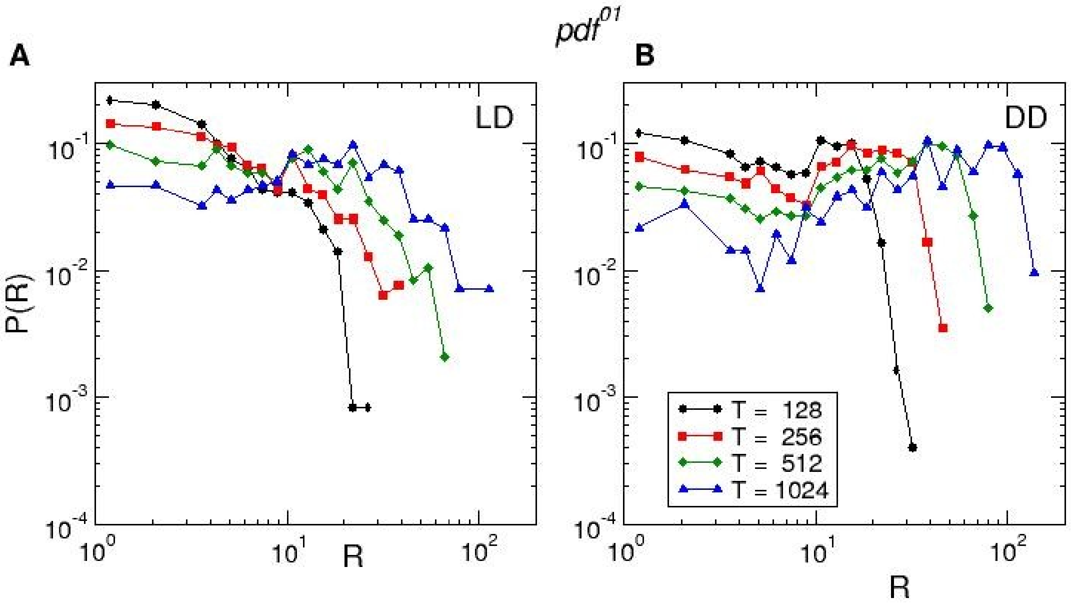

Figure S7: Activity rate distribution for *pdf^{01}* *(continued)*
Distribution of activity rates for ten *pdf^{01}* flies in LD conditions (A), and DD conditions (B), for four time windows T = 128, 256, 512 and 1024 seconds.

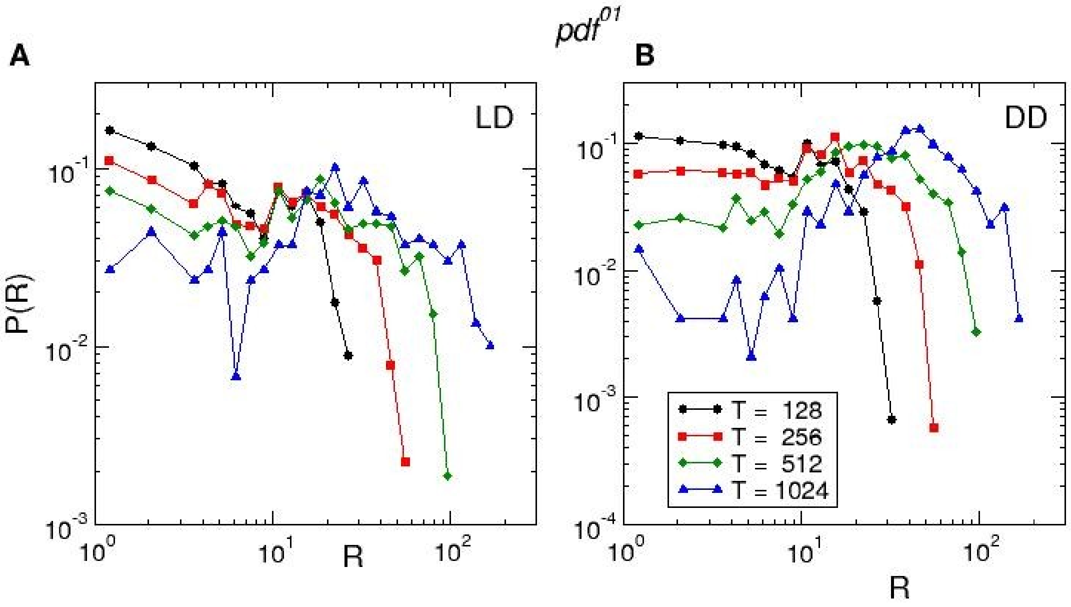

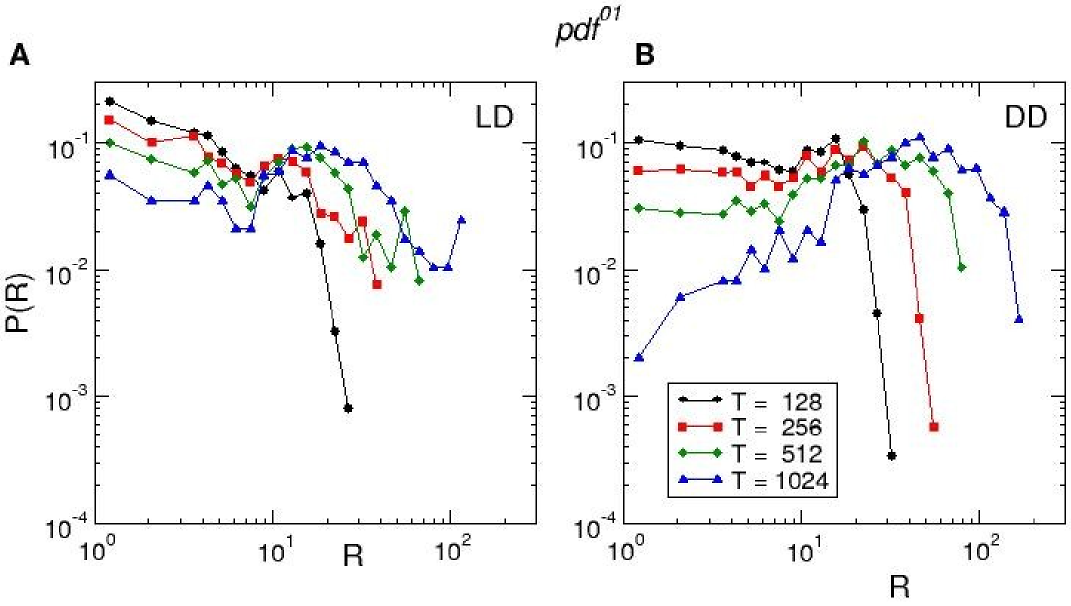

Figure S7: Activity rate distribution for *pdf*^{01} *(continued)*
Distribution of activity rates for ten *pdf*^{01} flies in LD conditions (A), and DD conditions (B), for four time windows T = 128, 256, 512 and 1024 seconds.
