# Supplementary material for: Role of the circadian clock in the statistics of locomotor activity in Drosophila
Source: PLoS One. 2018 Aug 23;13(8):e0202505. doi: 10.1371/journal.pone.0202505 (PMC6107170; doi:10.1371/journal.pone.0202505)
Supplement: S8 Fig — Distribution of activity rates for ten per01 flies in LD conditions (left column), and DD conditions (right column), for four time windows T = 128, 256, 512 and 1024 seconds. (PDF) [file pone.0202505.s008.pdf]

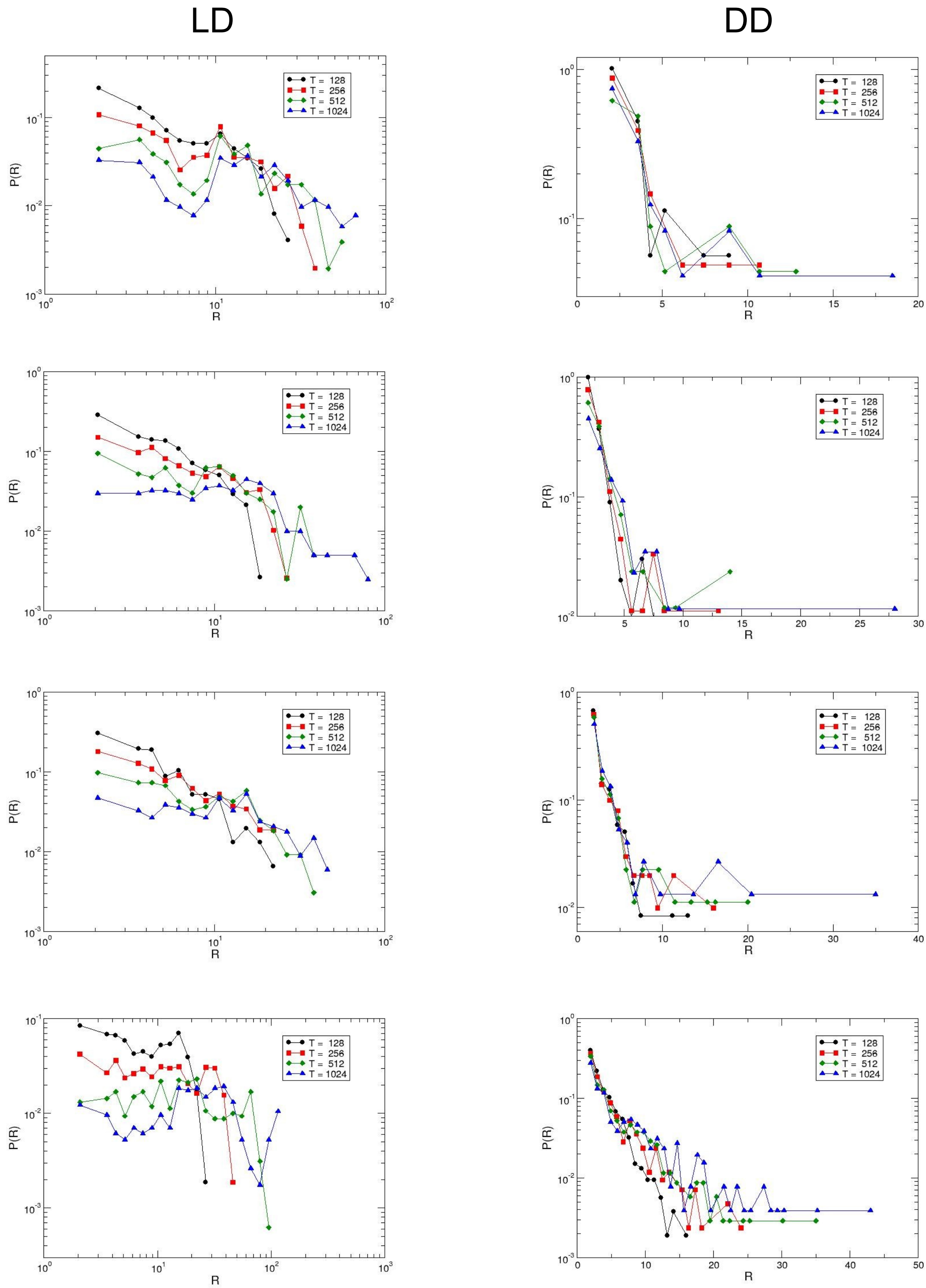

Figure S8: Activity rate distribution for *per^{01}*

Distribution of activity rates for ten *per^{01}* flies in LD conditions (left column), and DD conditions (right column), for four time windows T = 128, 256, 512 and 1024 seconds.

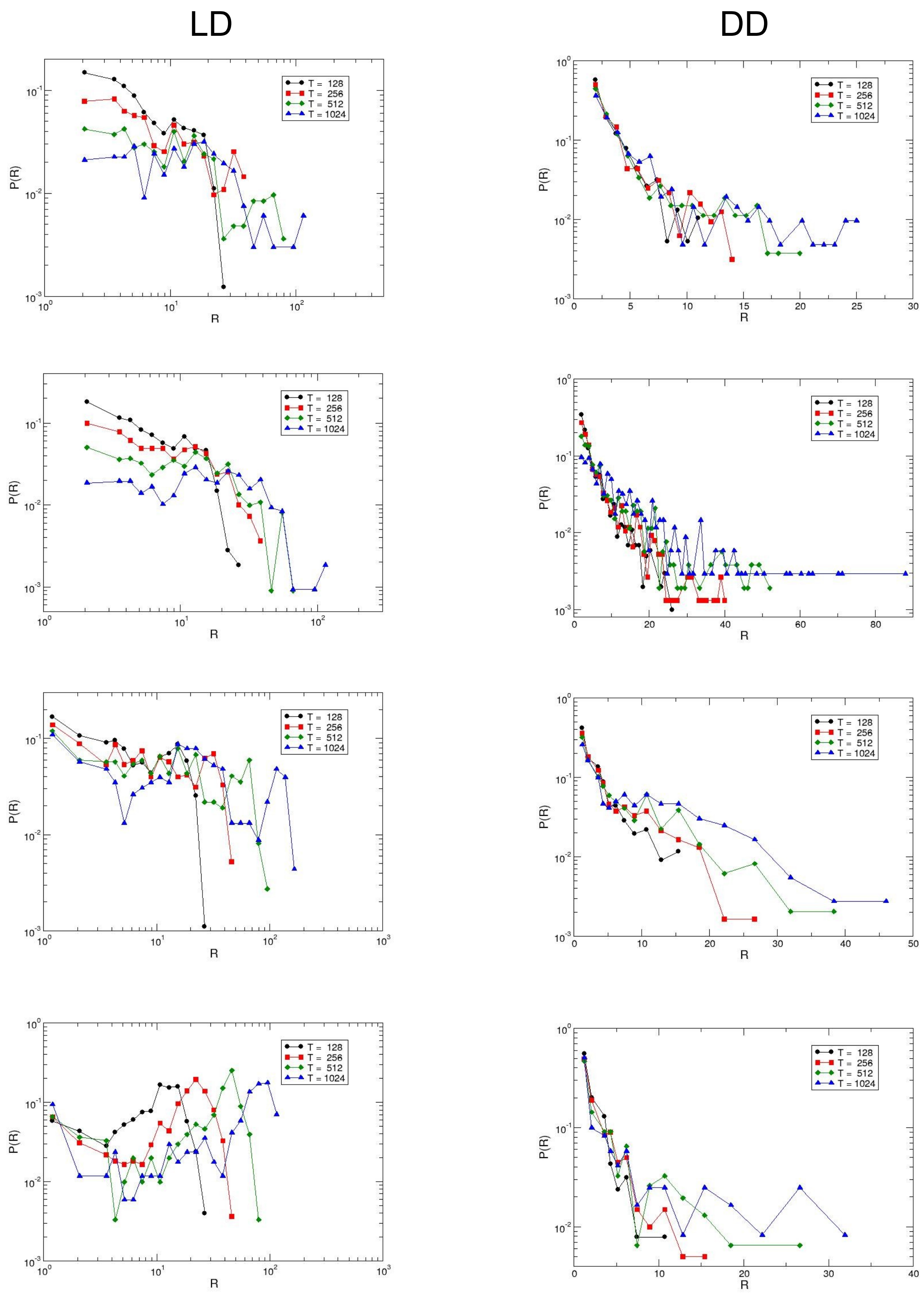

Figure S8: Activity rate distribution for *per*^{011} *(continued)*
Distribution of activity rates for ten *per*^{01} flies in LD conditions (left column), and DD conditions (right column), for four time windows T = 128, 256, 512 and 1024 seconds.

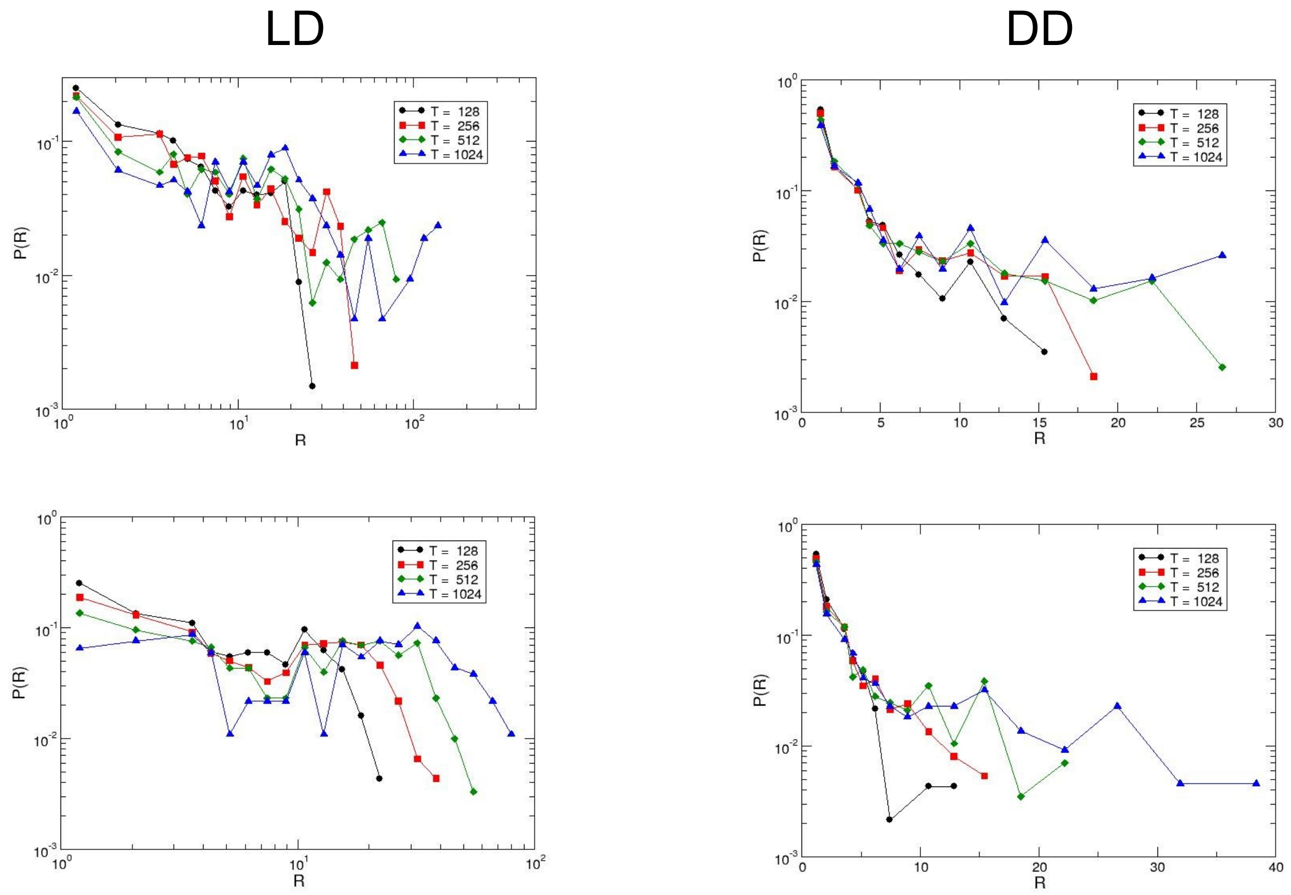

Figure S8: Activity rate distribution for *per*[01] *(continued)*
Distribution of activity rates for ten *per*[01] flies in LD conditions (left column), and DD conditions (right column), for four time windows T = 128, 256, 512 and 1024 seconds.
